# Sputum bacterial load and bacterial composition correlate with lung function and are altered by long-term azithromycin treatment in children with HIV-associated chronic lung disease

**DOI:** 10.1186/s40168-023-01460-x

**Published:** 2023-02-20

**Authors:** Regina E. Abotsi, Felix S. Dube, Andrea M. Rehman, Shantelle Claassen-Weitz, Yao Xia, Victoria Simms, Kilaza S. Mwaikono, Sugnet Gardner-Lubbe, Grace McHugh, Lucky G. Ngwira, Brenda Kwambana-Adams, Robert S. Heyderman, Jon Ø. Odland, Rashida A. Ferrand, Mark P. Nicol, The BREATHE study team

**Affiliations:** 1 Department of Molecular and Cell Biology & Institute of Infectious Diseases and Molecular Medicine, University of Cape Town, Cape Town, South Africa; 2 Department of Pharmaceutical Microbiology, School of Pharmacy, University of Health and Allied Sciences, Ho, Ghana; 3 International Statistics and Epidemiology Group, London School of Hygiene and Tropical Medicine, London, UK; 4 Division of Medical Microbiology, Department of Pathology, University of Cape Town, Cape Town, South Africa; 5 Marshall Centre, Division of Infection and Immunity, School of Biomedical Sciences, University of Western Australia, Perth, Australia; 6 Biomedical Research and Training Institute, Harare, Zimbabwe; 7 Computational Biology Group and H3ABioNet, Department of Integrative Biomedical Sciences, University of Cape Town, Cape Town, South Africa; 8 Department of Science and Laboratory Technology, Dar es Salaam Institute of Technology, Dar es Salaam, Tanzania; 9 Department of Statistics and Actuarial Science, Stellenbosch University, Stellenbosch, South Africa; 10 Malawi-Liverpool Wellcome Trust Clinical Research Programme, Blantyre, Malawi; 11 Department of Clinical Sciences, Liverpool School of Tropical Medicine, Liverpool, UK; 12 NIHR Global Health Research Unit on Mucosal Pathogens, Research Department of Infection, Division of Infection and Immunity, University College London, London, UK; 13 Department of Community Medicine, University of Tromsø, Tromsø, Norway; 14 International Research Laboratory for Reproductive Ecotoxicology (IL RET), The National Research University Higher School of Economics, Moscow, Russia; 15 School of Health Systems and Public Health, University of Pretoria, Pretoria, South Africa; 16 Clinical Research Department, London School of Hygiene and Tropical Medicine, London, UK

**Keywords:** Africa, Bacteriome, Microbiome, HIV, *Haemophilus*, *Moraxella*, Obliterative bronchiolitis, Adolescents, FEV1*z*

## Abstract

**Background:**

Long-term azithromycin (AZM) treatment reduces the frequency of acute respiratory exacerbation in children and adolescents with HIV-associated chronic lung disease (HCLD). However, the impact of this treatment on the respiratory bacteriome is unknown.

**Method:**

African children with HCLD (defined as forced expiratory volume in 1 s *z*-score (FEV1*z*) less than − 1.0 with no reversibility) were enrolled in a placebo-controlled trial of once-weekly AZM given for 48-weeks (BREATHE trial). Sputum samples were collected at baseline, 48 weeks (end of treatment) and 72 weeks (6 months post-intervention in participants who reached this timepoint before trial conclusion). Sputum bacterial load and bacteriome profiles were determined using 16S rRNA gene qPCR and V4 region amplicon sequencing, respectively. The primary outcomes were within-participant and within-arm (AZM vs placebo) changes in the sputum bacteriome measured across baseline, 48 weeks and 72 weeks. Associations between clinical or socio-demographic factors and bacteriome profiles were also assessed using linear regression.

**Results:**

In total, 347 participants (median age: 15.3 years, interquartile range [12.7–17.7]) were enrolled and randomised to AZM (173) or placebo (174). After 48 weeks, participants in the AZM arm had reduced sputum bacterial load vs placebo arm (16S rRNA copies/µl in log_10_, mean difference and 95% confidence interval [CI] of AZM vs placebo − 0.54 [− 0.71; − 0.36]). Shannon alpha diversity remained stable in the AZM arm but declined in the placebo arm between baseline and 48 weeks (3.03 vs. 2.80, *p* = 0.04, Wilcoxon paired test). Bacterial community structure changed in the AZM arm at 48 weeks compared with baseline (PERMANOVA test *p* = 0.003) but resolved at 72 weeks. The relative abundances of genera previously associated with HCLD decreased in the AZM arm at 48 weeks compared with baseline, including *Haemophilus* (17.9% vs. 25.8%, *p* < 0.05, ANCOM *ω* = 32) and *Moraxella* (1% vs. 1.9%, *p* < 0.05, ANCOM *ω* = 47). This reduction was sustained at 72 weeks relative to baseline. Lung function (FEV1*z*) was negatively associated with bacterial load (coefficient, [CI]: − 0.09 [− 0.16; − 0.02]) and positively associated with Shannon diversity (0.19 [0.12; 0.27]). The relative abundance of *Neisseria* (coefficient, [standard error]: (2.85, [0.7], *q* = 0.01), and *Haemophilus* (− 6.1, [1.2], *q* < 0.001) were positively and negatively associated with FEV1*z*, respectively. An increase in the relative abundance of *Streptococcus* from baseline to 48 weeks was associated with improvement in FEV1*z* (3.2 [1.11], *q* = 0.01) whilst an increase in *Moraxella* was associated with decline in FEV1*z* (-2.74 [0.74], *q* = 0.002).

**Conclusions:**

AZM treatment preserved sputum bacterial diversity and reduced the relative abundances of the HCLD-associated genera *Haemophilus* and *Moraxella*. These bacteriological effects were associated with improvement in lung function and may account for reduced respiratory exacerbations associated with AZM treatment of children with HCLD.

Video Abstract

**Supplementary Information:**

The online version contains supplementary material available at 10.1186/s40168-023-01460-x.

## Background

HIV-associated chronic lung disease (HCLD) is the most common chronic complication of HIV and accounts for more than 50% of all HIV-related mortality [1]. HCLD includes tuberculosis, chronic aspiration pneumonia and bronchiectasis [1]. Recently, in sub-Saharan Africa (Zimbabwe [2, 3], Malawi [4], South Africa [5, 6]), a novel HCLD phenotype, obliterative bronchiolitis, was detected at high prevalence (> 30%) among older children and adolescents living with HIV [3]. The symptoms of the condition include breathlessness, reduced exercise tolerance, fatigue, and chronic cough [2, 4]. The condition is associated with frequent acute respiratory exacerbations and hospitalisations, disruption in education due to absenteeism and reduced quality of life [3, 7].

Although the pathogenesis of this HIV-associated obliterative bronchiolitis is unknown, we speculate that it may be driven by the interplay between the dysregulated immune activation associated with HIV infection and the repeated respiratory infections these children experience whilst growing up in a setting of high infectious disease burden [8]. There are currently no evidence-based management guidelines for the condition. However, we have previously demonstrated that long-term azithromycin (AZM) treatment reduced the frequency of acute respiratory exacerbations and all-cause hospitalisations in children and adolescents with HCLD—the Bronchopulmonary Function in Response to Azithromycin Treatment for Chronic Lung Disease in HIV-infected Children (BREATHE) trial [7].

Long-term use of AZM has also been associated with improved lung function and survival and reduced frequency of acute respiratory exacerbation, antibiotic administration and hospitalisation in other chronic lung diseases (CLD) [9] such as cystic fibrosis [10], chronic obstructive pulmonary disease (COPD) [11], asthma [12], post-transplantation obliterative bronchiolitis [13] and bronchiectasis [14]. Mechanisms for these beneficial effects may include a combination of the antimicrobial, anti-inflammatory and immunomodulatory effects of AZM [9]. The direct antibacterial effect of AZM on pathogens may be enhanced by improved phagocytic competency of alveolar macrophages, which tend to be defective in CLDs [15, 16]. Additional benefits of AZM in CLDs include antiviral activity, improved ciliary function, interference with *Pseudomonas aeruginosa* biofilm formation, mitigation of airway mucus hypersecretion and promotion of pulmonary epithelial cell healing [9].

We previously observed that a *Haemophilus*, *Moraxella* or *Neisseria* (HMN)*-*dominant sputum bacteriome was associated with a 1.5-fold increased risk of HCLD, compared to a *Streptococcus* or *Prevotella*-dominant sputum bacteriome [17]. We also showed an association between HCLD and respiratory carriage of *Streptococcus pneumoniae* and *Moraxella catarrhalis* [18]. Huang et al. [19] observed that, in COPD, acute exacerbations were associated with an increase in the relative abundance of the phylum Proteobacteria (HMN belong to this phylum). Antibiotic treatment reduced this relative abundance and resulted in the resolution of symptoms. Although similar mechanisms may apply in HCLD, the effect of AZM on the respiratory microbiome in HCLD has not been studied. An insight into how AZM affects the HCLD respiratory microbiome and how this may mediate its beneficial effects will contribute towards understanding the pathogenesis of HCLD and facilitate targeted therapeutics.

In this study, we determined the impact of long-term AZM treatment (weekly treatment for 48 weeks) on the diversity and composition of the sputum bacteriome of Zimbabwean and Malawian children and adolescents with HCLD enrolled in the BREATHE trial. We also measured the persistence of bacteriome alterations 6 months post-treatment, which has not been previously investigated in other CLDs. Further, we assessed the association between bacteriome alterations and key clinical outcomes, including reduction in acute respiratory exacerbations, all-cause hospitalisations, and lung function. Finally, we investigated whether AZM altered the airway bacteriome in favour of HCLD-protective taxa.

## Methods

### Study design, participants, setting, eligibility, and intervention

This was a sub-study of the Bronchopulmonary Function in Response to Azithromycin Treatment for Chronic Lung Disease in HIV-infected Children (BREATHE) trial [7, 8] (clinicaltrials.gov identifier NCT02426112). The trial protocol [8] and its main findings [7] have been previously described. Briefly, children and adolescents aged 6–19 years with perinatally-acquired HIV infection taking antiretroviral therapy (ART) for a minimum of 6 months were recruited from outpatient HIV clinics in Blantyre, Malawi and Harare, Zimbabwe. HCLD was defined as forced expiratory volume in 1 s (FEV1) *z*-score (FEV1*z*) less than − 1.0 with no reversibility, i.e. < 12% improvement in FEV1*z* after 200 μg of salbutamol inhalation. FEV1z was calculated using the African American module of the Global Lung Function Initiative 2012 reference equations [20] validated among children in Zimbabwe by our team [21]. Exclusion criteria included acute respiratory symptoms, tuberculosis, pregnancy, hypersensitivity to AZM, prolonged QTc interval, impaired hearing or renal clearance. Participants were randomised to receive 48 weeks of once-weekly, weight-based doses of oral AZM [8]. Placebo arm participants received identical tablets without the active drug. For both trial arms, this was followed by a 6-month treatment-free period (to 72 weeks). Participant adherence was defined as “*not missing*, *on average*, *more than two of the 12 dispensed doses*, *as assessed by pill count*, *splitting time in the study into four 12-week periods*, *as per visit and study medication dispensing schedule*” [7]. All trial participants were analysed at 48 weeks*.* In this sub-study, only participants with sputum samples available from, at least, one of the three visits (baseline, 48 and 72 weeks) were included. Hospitalisation was defined as a period of stay in a hospital > 24 h. Acute respiratory exacerbation was defined as new or worsening respiratory symptoms, as assessed by a physician.

### Study outcomes

For this sub-study comparing AZM and placebo arms, the primary outcomes were within-participant change in bacteriome profiles (as defined by Aitchison distance, Bray-Curtis index and differentially abundant taxa) of the sputum samples from baseline to 48-week, 48- to 72-week, and from baseline to 72-week study visits. Secondary outcomes were (1) differences in sputum bacterial load and bacteriome at all visits between AZM and placebo arms; (2) associations between alpha and beta diversity metrics and selected covariates including drug adherence, age, sex, site, season of sampling, duration of ART, lung function, MRC dyspnoea score [22] and acute respiratory exacerbation; (3) differences in within-participant sputum beta diversity of participants who developed an adverse event during the study (acute respiratory exacerbation, hospitalisation or receipt of additional antibiotics other than study drug or cotrimoxazole) compared to those that did not; and (4) the differentially abundant taxa driving changes and differences in bacteriome profiles between trial arms and visits.

### Sample and data collection

Sputa were collected from participants at baseline, 48 weeks (end of treatment) and 72 weeks (6 months post-intervention in participants who reached this timepoint before trial conclusion). For participants who could not expectorate spontaneously, sputum was induced with hypertonic saline. Samples were stored in PrimeStore® Molecular Transport medium [Primestore] (Longhorn Vaccines & Diagnostics LLC, Bethesda, USA) immediately after collection, transported on ice and stored at – 80 °C at the study centres. This was followed by batch shipment on dry ice to Cape Town, South Africa, where samples were stored at − 80 °C until further processing. Clinical and socio-demographic data were collected at each visit using questionnaires administered by a study nurse and from participant hospital records.

### Nucleic acid extraction and purification

Sputum samples stored at − 80 °C were thawed, vortexed for 10 s and 450 μl transferred into ZR BashingBeadTM Lysis Tubes containing 0.5 mm beads (catalogue no. ZR S6002–50, Zymo Research Corp., Irvine, CA, USA). Mechanical lysis was done at 50 Hz for 5 min using the TissueLyser LTTM (Qiagen, FRITSCH GmbH, Idar-Oberstein, Germany). The lysate was centrifuged at 10,000 rpm (10,640 g) (Eppendorf F-45-30-11, Merck KGaA, Darmstadt, Germany) for 2 min, and 250 μl of the supernatant was loaded onto a QIAsymphony® SP instrument (Qiagen, Hombrechtikon, Switzerland). Nucleic acid was purified using the DSP Virus/Pathogen Mini Kit® (catalogue no. 937036, Qiagen GmbH, Hilden, Germany) and eluted into 70 μl. We included bacterial mock community cells (catalogue no. ZR D6300, Zymo Research Corp., Irvine, CA, USA) as template extraction controls on each of the four plates of the three sequencing runs. We also included neat Primestore and Milli-Q® ultrapure water (exposed to UV for 30 min prior to use) (Millipore Sigma, Burlington, MA, USA) as no-template extraction controls.

### 16S rRNA library preparation and amplicon sequencing

The total bacterial load (16S rRNA gene copies) of each extracted sample was determined using a previously described qPCR [23]. Gene copy numbers were extrapolated from standard curves derived from *Escherichia coli* strain JM109 DNA (catalogue no. ZR E2006, Zymo Research Corp., Irvine, CA, USA), using genome size (4.60E+06) and number of 16S rRNA gene copies (seven). For bacteriome analysis, the V4 hypervariable region of the 16S rRNA gene underwent two-step amplification as previously described [24] with the following modifications: 5 µl of DNA was used as template for each step of the PCR with a final concentration of 8pm loaded on the Illumina MiSeq instrument using MiSeq Reagent Kit V3 600 cycles (Illumina Inc., San Diego, USA). A custom primer set was used for sequencing (Read1_seq Primer Sequence 5′-3′–TATGGTAATTGTGTGCCAGCHGCYGCGGTAA, Read2_seq Primer Sequence 5′-3′–AGTCAGTCAGCCGGACTACHVGGGTWTCTAAT, Index_seq Primer Sequence 5′-3′ ATTAGAWACCCBDGTAGTCCGGCTGACTGACT). We also included a bacterial mock community DNA standard (catalogue no. ZR D6305, Zymo Research Corp., Irvine, CA, USA) as sequencing controls and no template extraction controls on all plates in each run. Randomly selected samples were repeated within and between sequencing runs to assess within and between-run reproducibility. Samples from the same participant were all processed on the same plate to avoid batch effects. Each run included a balanced number of samples from both trial arms and from both study sites.

### Bioinformatics analysis of 16s rRNA amplicon sequence data

The quality of demultiplexed raw sequence reads was assessed using the FastQC [25] and MultiQC [26] tools. Per base sequence quality scores were uniformly higher than the threshold Phred score of 20. The DADA2 pipeline [27] (wrapped in the Nextflow algorithm [28]) was used to filter and trim reads, infer amplicon sequence variants (ASVs), and assign taxonomy to ASVs. Default parameters were applied for all DADA2 functions unless expressly mentioned. Briefly, forward reads were trimmed and truncated at 24 and 248 bases, and reverse reads were trimmed and truncated at 25 and 235 bases. The minimum read length after trimming and truncation was set to 250 bases. Sequencing reads were dereplicated and pooled, and ASVs were inferred for each sample via the DADA2 sample inference algorithm and the estimated error model. Denoised sequences were generated by merging forward and reverse reads with overlap length set to 20 and allowing for no mismatches in the overlap region. Chimeric sequences were identified through exactly reconstructing them by combining a left-segment and a right-segment of more abundant sequences and then were removed from the ASV table of denoised merged sequences. Taxonomy was assigned to each ASV using the SILVA version 138 [29] species classifier implementation for DADA2 [27]. Phylogenetic alignment was done using *AlignSeqs* in the DECIPHER [30] package whilst a neighbour-joining phylogenetic tree was constructed using functions in the PHANGORN [31] package. The 16S rRNA gene sequencing datasets were submitted to the National Centre for Biotechnology Information (NCBI) Sequence Read Archive (SRA) repository (Accession number PRJNA769290).

### Quality control and in silico correction of contamination

Both experimental work and data analysis plan were completed before unblinding of study investigators. We used a previously published in-silico quality control approach to minimise experimental error as outlined below [32]. Spurious ASVs, defined as ASVs with < 5 reads across all sequenced biological specimens and no-template control, were removed. We assessed nucleic acid extraction and sequencing efficiency by comparing the mock bacterial community extraction and sequencing controls to the manufacturer profiles. Next, we assessed reproducibility within and between runs by comparing bacteriome profiles of within- and between-run repeats. To determine whether the bacteriome profile of the low biomass samples was background noise from the storage medium (Primestore) or amplicon contamination, we assessed clustering of these samples with the no-template controls (Primestore) on a log-ratio biplot and Principal Coordinates Analysis (PCoA) plot. Next, biological specimens with 16S rRNA gene copy numbers ≤ 500/μl were removed, as we and others have shown that these low biomass specimens produce poorly reproducible sequencing profiles [32, 33]. We used the *isContaminant* function embedded in the DECONTAM [34] package in R to identify potential contaminants using sequence data from biological specimens and no-template controls (Primestore). The maximum proportions of each identified contaminant in the no-template controls were subtracted from the biological specimens. Detailed quality control and decontamination steps and results are described in the supplementary material (Supplementary Table S1 and S2, Supplementary Figure S1-S17).

### Statistical analysis

Statistical analysis was performed using R statistical software (version 4.0.4). Categorical variables were reported as frequency distributions and continuous variables as medians with interquartile ranges (IQRs) if non-parametric or means with standard deviations (SD) if otherwise. Normality distribution of continuous variables was assessed using Shapiro Wilk test. Between trial arm comparisons of continuous outcomes were done using Mann-Whitney *U* or Student *t*-test where appropriate. Fisher’s exact test or chi-square test were used to compare categorical variables between arms where appropriate. We used Wilcoxon signed-rank matched-pairs test to compare continuous outcomes between visits within trial arms. Correction for multiple testing was with Benjamin/Hochberg (cut off = 0.05). Amplicon sequence variants (ASV) were merged to the genus level, and 0.5% prevalence filtering was applied to samples before analysis. The number of ASVs in a single sample represented bacterial taxonomic richness. Shannon’s index [35] was used to characterise richness and evenness (alpha diversity).

Beta diversity was determined for intra-individual differences in samples from different visits or inter-individual differences in samples collected at the same visit. Beta diversity was determined using Euclidean distances of centered log-ratio (CLR) transformed ASV counts (Aitchison distance) after applying a pseudo count of one or by Bray-Curtis dissimilarity index [36] on unrarefied ASV counts, all implemented using *distance* functions in the PHYLOSEQ R package. Principal coordinates analysis (PCoA) plots were used for the visualisation of beta diversity. We used permutational multivariate analysis of variance (PERMANOVA) [37] implemented using the *adonis2* function in the VEGAN [38] R package to compare beta diversity between visits or trial arms. PERMANOVA tests were adjusted for age, sex, site, ART regimen, MRC dyspnoea score, FEV1*z*, cotrimoxazole prophylaxis, previous tuberculosis treatment and previous admission for chest problems within 12 months prior to enrolment. Permutational analyses of multivariate dispersions (PERMDISP) (implemented using *betadisper* function in VEGAN) was used to assess the homogeneity of dispersion between visits and trial arms.

We used ten methods for detecting differentially abundant taxa. These are Wilcoxon signed-rank test on data after the following normalisation methods–total sum scaling, variance stabilising transformation, and centred log transformation after applying a pseudo count of one, ANCOM2 [39], Aldex2 [40], DESeq2 [41], ANCOM-BC [42], Corncob [43], MaasLin2 [44] with total sum scaling and log transformation and MaasLin2 [44] with centered log transformation after applying a pseudo count of one. We reported the findings for all of these methods; however, for our primary analysis we have used the results of ANCOM2 [39] [on unrarefied data with false discovery rate (FDR) Benjamini/Hochberg correction (cut off = 0.05)] because Nearing et al. [45] have recently demonstrated that this method is one of the most consistent approaches and agrees best with the intersect of results from different approaches. The relative contribution of each taxon to overall dissimilarity was measured using SIMPER analysis on the Bray-Curtis distances between samples.

To determine the association between sputum bacterial load (log_10_ transformed 16S rRNA gene copies) or alpha diversity (Shannon index) and clinical and socio-demographic factors, we used linear mixed effect models (LME) via the LME4 and LMERTEST R packages. For each LME, we included visit and the interaction term visit-versus-trial-arm [46] as fixed effects and the individual participant identifier as a random effect. We included in a stepwise manner, adherence, age, site, sex, season of sampling, Medical Research Council (MRC) dyspnoea score, body mass index (BMI), weight-for age-*z*-score, height-for age-*z*-score, BMI-for age-*z*-score, ART regimen, duration of ART, HIV viral load suppression, cotrimoxazole prophylaxis, previous tuberculosis treatment or previous hospitalisation for chest problems 1 year prior to study enrolment, forced vital capacity (FVC), forced vital capacity *z*-score (FVC*z*), FEV1*z*, FEV1 percentage predicted (FEVpcpred), FVC percentage predicted (FVCpcpred) and FEV1/FVC*z* as covariates in the models. Continuous outcome variables included in the models were log-transformed where necessary to satisfy model assumptions. Adherence to AZM, age, site, sex, season of sampling, MRC dyspnoea score, HIV viral load suppression (baseline values were used as values at 72 weeks were unavailable), cotrimoxazole prophylaxis, previous tuberculosis treatment or previous hospitalisation for chest problems 1 year prior to study enrolment, ART regimen and duration were selected a priori to be adjusted for. Any other variable identified as an independent predictor in the univariate analysis (*p* < 0.05) was also included in the multivariate LME model. The following were excluded from the multivariate model because of co-linearity: BMI-for-age *z*-score and weight-for-age (colinear with height-for-age) and CD4 count (colinear with viral load suppression). We also assessed the association between bacterial genera and FEV1z, FVCz and adverse events (hospitalisation, additional antibiotic use and acute respiratory exacerbation during intervention) using a linear mixed effect model implemented with MaasLin2 [44]. FEV1z, visit, interaction between trial arm and visit were included as fixed effects and participant as random effect with ASV counts total sum scaled and converted to 100% and without log transformation. We also used linear regression implemented with MaasLin2 [44] to investigate the association between the within-participant change in FEV1*z* and FVC*z* over time in each trial arm (fixed effects) and (i) within-participant change in the percentage relative abundance of the five bacterial genera of interest (*Streptococcus*, *Prevotella*, *Haemophilus*, *Neisseria* and *Moraxella*) and (ii) within-participant (between visit) beta diversity (Aitchison distance). The results of the regressions were reported as beta coefficients and standard error of the models.

## Results

### Participant characteristics

The BREATHE trial took place between June 2016 and September 2019. The socio-demographic and clinical data have been previously published [7]. Briefly, 347 children and adolescents were randomised, 173 to the AZM arm and 174 to the placebo arm. The median age of the participants at baseline was 15.3 years, interquartile range [12.7–17.7] and 49% (170/347) were female. The participants in the AZM arm were younger and had been more frequently treated for tuberculosis than those in the placebo arm. Other characteristics did not significantly differ between arms. Almost all participants (90%, 313/347) were receiving long-term cotrimoxazole prophylaxis. The baseline clinical, socio-demographic and microbiological features of the participants are presented in Table 1. During the intervention, fewer participants in the AZM arm experienced acute respiratory exacerbations (16, 9.2%) and hospitalisations (2, 1.2%) compared to those in the placebo arm (exacerbations: 30, 17.2%, hospitalisation, 9, 5.2%) [7].

**Table 1:** Characteristics of study participants at baseline

Characteristics	AZM arm ***N*** = 173	Placebo arm ***N*** = 174
**Demographic characteristics**		
Age in years, median (IQR)	14.7 (12.6–16.8)	15.8 (13.0–18.1)
Female sex, no. (%)	80 (46.2)	90 (51.7)
Currently in school, no. (%)^*a*^	146 (84.5)	139 (79.9)
Site: Zimbabwe, no. (%)	120 (69)	121 (70)
**HIV characteristics**		
Duration on ART in years, median (IQR)	5.9 (3.8–9.0)	6.4 (3.9–8.2)
HIV viral load suppression, < 1000 copies/ml, no. (%)	102 (59.0)	94 (54.0)
CD4 count category, >200 cells/μl, no. (%)	157 (91)	156 (89.7)
Antiretroviral regimen NNRTI, no. (%)^a^	127 (73)	131 (75)
**Lung function**		
FEV1 *z*-score, median (IQR)	-1.94 (-2.5, -1.4)	-2.0 (-2.4, -1.5)
**Clinical characteristics**		
Underweight, no. (%)^*b*^	98 (56.6)	83 (47.7)
Stunted, no. (%)^*c*^	95 (54.9)	80 (46.0)
History of TB, no. (%)^a^	58 (33.6)	39 (22.4)
Admitted for chest problems in last year, no. (%)	3 (1.7)	3 (1.7)
MRC dyspnoea score		
1	89 (51)	96 (55)
2	64 (37)	62 (36)
3	12 (7)	11 (6.3)
4	7 (4)	4 (2.3)
5	1 (1)	1 (1)
Cotrimoxazole prophylaxis^a^	157 (92)	156 (90)
^**d**^**Sputum culture**		
*Streptococcus pneumoniae, *no. (%)	43 (26)	38 (23)
*Staphylococcus aureus, *no. (%)	51 (31)	46 (28)
*Haemophilus influenzae, *no. (%)	4 (2)	7 (4)
*Moraxella catarrhalis, *no. (%)	15 (9)	15 (9)
Any of the four bacteria above, no. (%)	82 (49)	79 (48)
**Sputum total bacterial load**		
Sputum bacterial count (16S copy numbers in log_10_), median (IQR)	5.0 (4.45–5.45)	4.93 (4.41–5.54)
**Sputum bacteriome alpha diversity**		
Shannon-Wiener diversity, median (IQR)	3.0 (2.5–3.3)	3.0 (2.4 –3.4)
Observed Taxon richness, median (IQR)	29 (22–35)	29 (23–36)
^**e**^**Sputum bacteriome within-arm beta diversity**		
Aitchison distance, median (IQR)	34.2 (30.9–37.5)	35.0 (32.0–38.3)
Bray-Curtis index, median (IQR)	0.69 (0.57–0.83)	0.71 (0.59–0.84)

^a^Missing value: currently attending school – *n* = 1 AZM arm; antiretroviral regimen – *n* = 1, placebo arm; history of TB – *n* = 1, AZM arm; cotrimoxazole prophylaxis – *n* = 2, AZM arm

^b^Weight-for-age-*z*-score < − 2

^c^Height-for-age-z-score < –2, FEV1: forced expiratory volume in 1 s; participants with missing responses are excluded from that variable

^d^Number of sputum samples is 166 (AZM) and 164 (placebo)

*IQR* interquartile range, *NNRTI* non-nucleoside reverse transcriptase inhibitor

^e^Compared using PERMANOVA test

### Number of samples

**Fig. 1:**
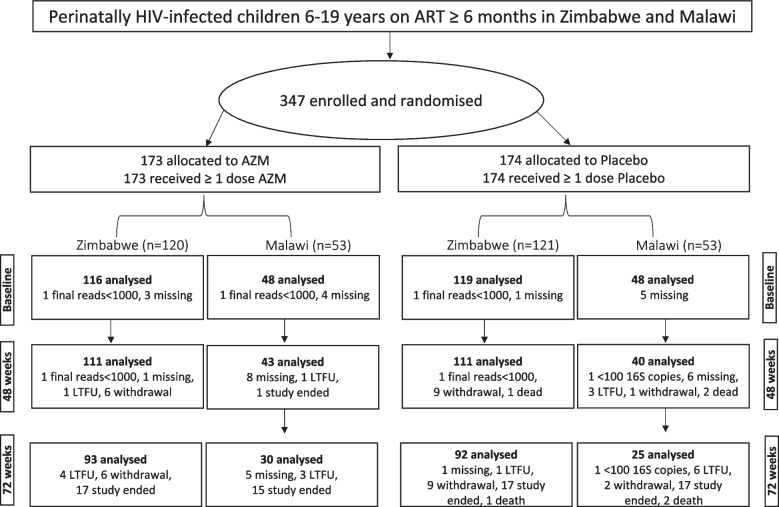
Flow chart of the number of samples collected from each study site at each visit and the numbers included and excluded from final analysis. LTFU, loss to follow up; 16S copies, 16S rRNA copies

Ninety-nine percent of all samples (869/875) were spontaneously expectorated, whilst the remainder were induced (3 samples in each study arm). Nineteen percent of participants (66/347) were not followed up at 72 weeks due to logistical limitations. Details of the number of samples collected from each study site and at each visit and the numbers included and excluded are summarised in Fig. 1.

### AZM reduced sputum bacterial load among participants in the intervention arm

**Fig. 2:**
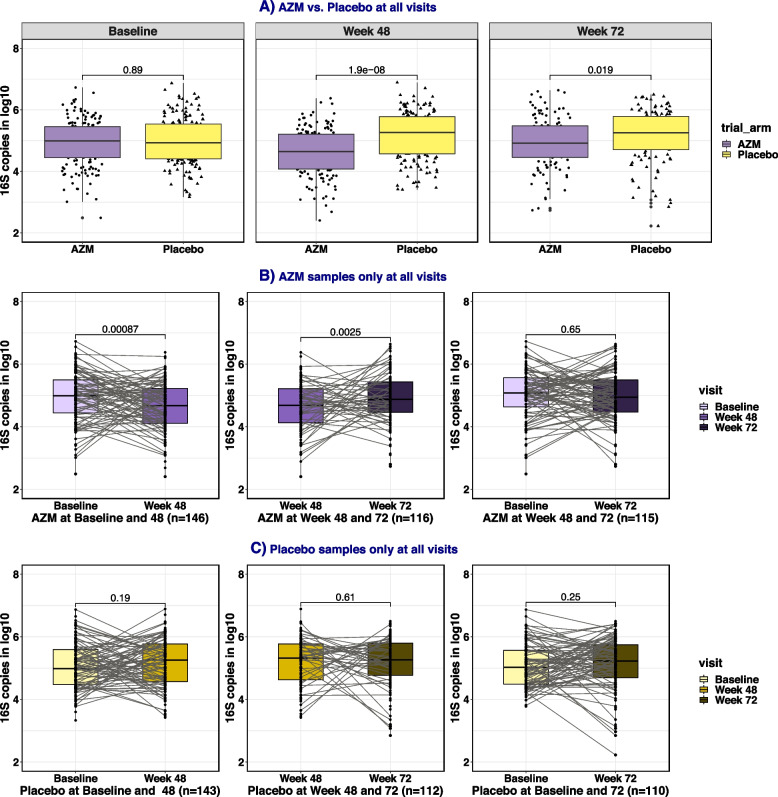
Boxplot of bacterial load between trial arms at each visit (**A**) and between visits within AZM arm only (**B**) and placebo arm only (**C**). 16S rRNA gene copy number, used as proxy for total bacterial load, was measured with qPCR. The between arm comparisons were implemented using Wilcoxon signed rank test for unpaired samples whilst within-arm comparisons used Wilcoxon signed rank test for paired samples

The median bacterial load (log_10_16S rRNA gene copies/μl) of the 875 samples was similar between the two arms at baseline [median (IQR) AZM, 5.0 (4.45–5.45) vs placebo 4.93 (4.41–5.54), *p* = 0.89] but was lower in AZM than placebo at both the 48-week [median (IQR) AZM, 4.65 (4.08–5.21) vs placebo 5.27 (4.57–5.78), *p* < 0.0001] and 72-week visits [median (IQR) AZM, 4.92 (4.46–5.48) vs placebo 5.26 (4.71–5.79), *p* = 0.019] (Fig. 2A). Within the AZM arm, median 16S rRNA gene copy number at 48 weeks [4.65 (4.08–5.21)] was lower than at baseline [5.0 (4.45–5.45), *p* < 0.001] and at 72 weeks [4.92 (4.46–5.48), *p* = 0.003] (Fig. 2B). Bacterial load remained similar across visits in the placebo arm (Fig. 2C).

We used LME, Table 2 (Table S3), to explore associations between bacterial load (16S rRNA copies) and clinical and socio-demographic characteristics. Although bacterial load remained constant in the placebo arm, it declined significantly at 48 weeks compared to baseline in the AZM arm (adjusted beta coefficient and 95% confidence interval: − 0.5 log_10_ copies per µl [− 0.63; − 0.29]). Participants from the Zimbabwean study site on average had a higher bacterial load than those from Malawi (0.3 log_10_ copies per µl [0.11; 0.49]) at any visit. Better lung function was associated with lower bacterial load. Specifically, a one unit increase in FEV1*z* was associated with 0.1 log_10_ copies per µl [0.02–0.2] decrease in bacterial load at any visit. Participants who had received treatment for tuberculosis before enrolment had higher bacterial loads (0.2 log_10_ copies per µl [0.03–0.32]) at any visit than those who were not previously treated.

**Table 2:** Variables associated with (1) bacterial load (16S rRNA copies) and (2) Shannon diversity indices in multivariate linear mixed effect models

				Bacterial load (16S rRNA copies)		Shannon diversity index	
^*^Variable	Levels	^**a**^Number of observations (***n*** = 875)	Participants (***n*** = 346)	^**1**^Adjusted co-efficient (95% CI)	^**1**^***p*** value	^**2**^Adjusted co-efficient (95% CI)	^**2**^***p*** value
Visit	Placebo at week 48	150 (17.1%)	150 (43.4%)	Reference		Reference	
	AZM at week 48	154 (17.6%)	154 (44.5%)	**− 0.46 [− 0.63; − 0.29]**	< 0.0001	**0.25 [0.07; 0.42]**	0.01
	Placebo at week 72	117 (13.4%)	117 (33.8%)	Reference		Reference	
	AZM at week 72	123 (14.1%)	123 (35.5%)	− 0.19 [− 0.38; 0.0]	0.051	**0.2 [0.01; 0.40]**	0.04
Site	Malawi	233 (26.6%)	106 (30.6%)	Reference		Reference	
	Zimbabwe	642 (73.4%)	240 (69.4%)	**0.3 [0.11; 0.49]**	0.003	**0.27 [0.06; 0.47]**	0.01
Medical Research Council dyspnoea score at baseline	1	479 (54.7%)	184 (53.2%)			Reference	
	2	316 (36.1%)	126 (36.4%)			**0.26 [0.1; 0.42]**	0.04
	3	53 (6.1%)	23 (6.6%)			0.16 [− 0.18; 0.49]	
	4	23 (2.6%)	11 (3.2%)			0.25 [− 0.21; 0.71]	
	5	4 (0.5%)	2 (0.6%)			0.52 [− 1.05; 2.09]	
Forced expiratory volume in 1 s (FEV1) *z*-score (FEV1z)		862 (98.5%)	346 (100%)	**− 0.09 [− 0.16; − 0.02]**	0.02	**0.19 [0.12; 0.27]**	< 0.001
Ever treated for tuberculosis before enrolment	No	609 (69.8%)	248 (71.9%)	Reference		Reference	
	Yes	263 (30.2%)	97 (28.1%)	**0.17 [0.03; 0.32]**	0.02	**− 0.19 [− 0.34; − 0.04]**	0.02

^a^The difference between the number of observations and the total (875) represent number of missing observations for that variable

^1^The estimate of coefficient with 95% confidence intervals and *p* values were obtained from multivariate linear mixed effect model with participant included as a random effect, trial arm, visit and trial arm: visit interaction term and selected variables ( visit, site, Medical Research Council dyspnoea score at baseline, forced expiratory volume in 1 s (FEV1) *z*-score (FEV1z), previous tuberculosis treatment prior to enrolment, age group at baseline, adherence, site, sex, season of sampling, viral load suppression at baseline, height-for-age *z*-score, cotrimoxazole prophylaxis at baseline, previous admission to chest problems in the year preceding enrolment, duration of ART at baseline) as explanatory variables and 16S rRNA copies of the sputum samples as dependent variable

^2^The estimate of coefficient with 95% confidence intervals and p values were obtained from multivariate linear mixed effect model with participant included as a random effect, trial arm, visit and trial arm: visit interaction term and selected variables ( visit, Medical Research Council dyspnoea score at baseline, forced expiratory volume in 1 s (FEV1) *z*-score (FEV1z), previous tuberculosis treatment prior to enrolment, age group at baseline, adherence, site, sex, season of sampling, viral load suppression at baseline, height-for-age *z*-score, any events during intervention, cotrimoxazole prophylaxis at baseline, previous admission to chest problems in the year preceding enrolment, duration of ART at baseline) as explanatory variables and Shannon indices of the sputum samples as dependent variable. Any event refers to either acute respiratory exacerbation; additional antibiotics other than interventional drug or cotrimoxazole; or hospitalisation during intervention. Forced expiratory volume in 1 s (FEV1)/forced vital capacity *z*-score, forced vital capacity *z*-score (FVCz), FEV1 percentage predicted (FEVpcpred), FVC percentage predicted (FVCpcpred) were excluded from the final models because of collinearity with FEV1 *z*-score. Viral load and CD4 counts were excluded from the final models because data was not collected at 72 weeks, the values at baseline were used instead. Weight-for-age *z*-score, body mass index for age *z*-score were excluded from the final models because of collinearity with height-for-age-*z*-score

^3^Any event refers to either acute respiratory exacerbation; additional antibiotics other than interventional drug or cotrimoxazole; or hospitalisation during intervention

*Full table with coefficients and 95% confidence intervals from univariate linear mixed effect models are included supplementary materials

### Association between AZM treatment, clinical and socio-demographic characteristics and sputum alpha diversity

The median number of sequences per sample was 36,456 (IQR: 31,392–42,086) which reduced to 16,969 (IQR: 13,302–19,902) with a total of 1665 ASVs, after quality control and in silico decontamination steps. We used LME, Table 2 (Table S4), to explore associations between within-sample diversity (Shannon alpha diversity index) and clinical and socio-demographic characteristics. Although, the Shannon alpha diversity in the AZM arm remained unchanged (Figure S18B), it was significantly higher than placebo at 48 weeks (0.25 [0.07; 0.42]) and 72 weeks (0.2 [0.01; 0.40]). Participants from the Zimbabwean study site, on average, also had a higher Shannon diversity index than those from Malawi (0.27 [0.06; 0.47]) at any visit. Also, participants with MRC dyspnoea score of two had on average 0.26 units [0.1; 0.42] higher Shannon diversity index (higher sputum bacteriome alpha diversity) than participants with a score of one. Participants with higher FEV1*z* at any visit had a more diverse sputum bacteriome (higher Shannon index) compared to those with lower scores (0.19 [0.12; 0.27]). Finally, participants who were previously treated for tuberculosis before enrolment had, on average, 0.2 units [–0.34; –0.04] lower Shannon diversity index at any visit than those who were never treated.

### AZM treatment resulted in changes in the overall bacteriome profile

**Fig. 3:**
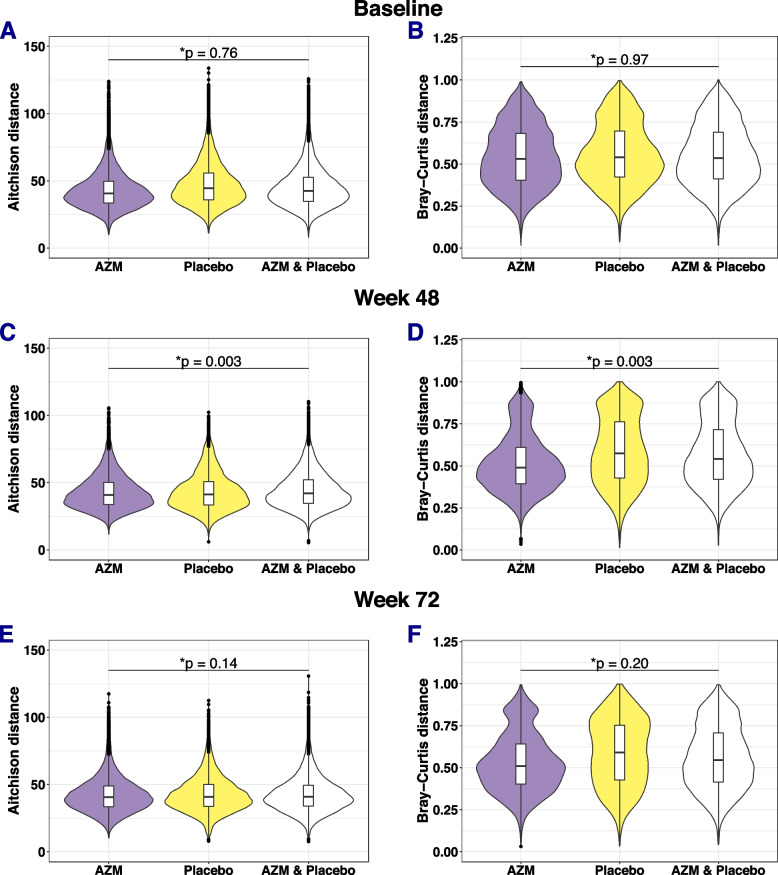
Violin boxplot comparing two beta diversity metrics between trial arms at 0, 48 and 72 weeks. **A** Comparison of AZM and placebo at baseline using Aitchison distance. **B** Comparison of AZM and placebo at baseline using Bray-Curtis distance on unrarefied ASV counts. **C** Comparison of AZM and placebo at 48 weeks using Aitchison distance. **D** Comparison of AZM and placebo at 48 weeks using Bray-Curtis distance on unrarefied ASV counts. **E** Comparison of AZM and placebo at 72 weeks using Aitchison distance. **F** Comparison of AZM and placebo at 72weeks using Bray-Curtis distance on unrarefied ASV counts. Asterisk (*) symbol indicates the following: *p* values were adjusted using BH correction. The first two violin boxplots of each figure shows the distribution of the within group distances in the AZM and placebo arms respectively. The third violin boxplots of each figure shows the distribution of the between group distance between AZM and placebo arms. The horizontal line in the middle of the box is the median. The box presents interquartile range. The whiskers show 95% confidence interval. The shape of the violin display frequencies of values

Beta diversity among samples from participants in the AZM arm between baseline and 48 weeks [PERMANOVA test, on Aitchison distance *p* = 0.004, Bray-Curtis, *p* = 0.002], or between 48 weeks and 72 weeks [Aitchison distance, *p* = 0.004, Bray-Curtis, *p* = 0.002], was greater than between samples within the same visit, showing within-participant changes in overall community structure across visits (Supplementary Figures S19). There was no difference in dispersion of samples between visits (PERMDISP, *p* > 0.05). In contrast, no difference in beta diversity between visits, compared to within visits, was observed within the placebo arm (Figures S20). At baseline, between-arm beta diversity did not differ significantly from within arm beta diversity in either study arm [PERMANOVA test on Aitchison distance, (*p* = 0.76), Bray-Curtis, (*p* = 0.97), Figure S21A, B, Fig. 3A, B]. However, at 48 weeks, overall bacterial composition differed between the two trial arms, with greater diversity between arms than within arms [PERMANOVA test on Aitchison distance, (*p* = 0.003), Bray-Curtis, (*p* = 0.003) Figure S21C, D and Fig. 3]. A dispersion effect was absent when considering Aitchison distance (PERMDISP *p* = 0.85) but was present when considering Bray-Curtis distance (PERMDISP *p* = 0.002), with greater dispersion in the placebo than in the AZM arm (Fig. 3). The difference in overall bacterial composition was not evident at the 72-week visits [PERMANOVA test on Aitchison distance, (*p* = 0.14), Bray-Curtis, (*p* = 0.2), Figure S21E, F and Fig. 3].

**Fig. 4:**
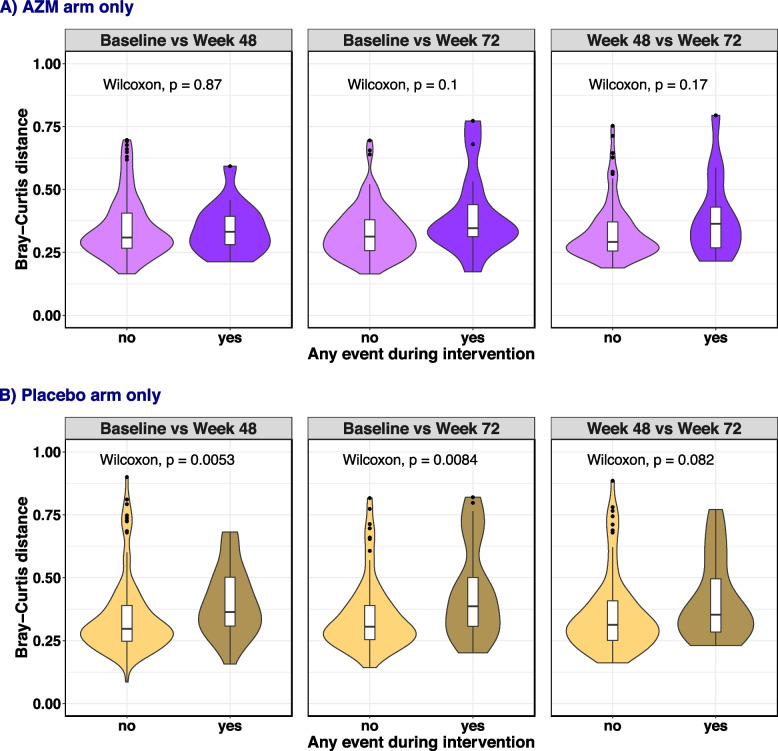
Violin boxplot comparing the within-participant change in Bray-Curtis distance on CLR transformed ASV counts from baseline to 48 weeks, baseline to 72 weeks and 48 weeks to 72 weeks between participants who developed acute exacerbation, hospitalised or were administered additional antibiotics (yes) with those who did not (no), in the AZM arm (**A**) and placebo arm (**B**). Wilcoxon test was used for all comparisons. The horizontal line in the middle of the box is the median. The box presents interquartile range. The whiskers show 95% confidence interval. The shape of the violin display frequencies of values

Within the placebo arm, the median within-participant Bray-Curtis distance between samples from participants who had an adverse event (acute exacerbation of respiratory illness, hospitalisation or required additional antibiotics, *n* = 115) was higher than between samples from participants who did not have an adverse effect (*n* = 35), when comparing baseline to 48 weeks (Kruskal Wallis test, *p* = 0.005), or baseline to 72 weeks (Kruskal Wallis test, *p* = 0.008) (Fig. 4B). Within the AZM arm, fewer participants developed an adverse event (*n* = 16), and there were no significant differences in within-participant Bray-Curtis distance across any of the timepoints when comparing with participants who did or did not have an adverse event (Fig. 4A).

### Composition of taxa within the sputum bacteriome of participants

Five phyla accounted for 99.2% of the mean relative abundances of all bacteria, Proteobacteria (53.4%), Firmicutes (23.4%), Bacteroidetes (14.2%), Fusobacteria (5.2%) and Actinobacteria (3.0%) (Figure S22). At genus level, the 10 most abundant genera at baseline included *Haemophilus* (25.5%), *Neisseria* (18.8%), *Streptococcus* (15.8%), *Prevotella* (9.5%), *Veillonella* (3.9%), Fusobacterium (3.2%), *Alloprevotella* (2.9%), *Porphyromonas* (2.7%), *Moraxella* (2.5%) and *Leptotrichia* (2.3%) (Figure S23).

**Fig. 5:**
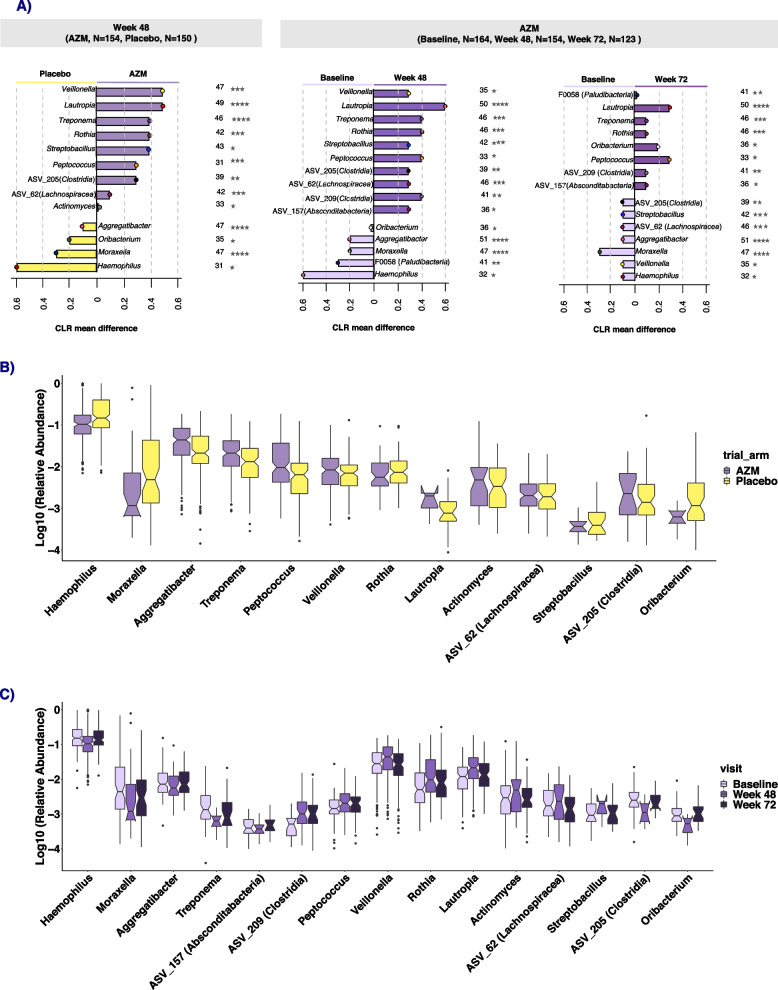
Analysis of composition of microbiomes (ANCOM2) differential abundance testing for samples at each visit and between trial arms. Taxa identified as differentially abundant between trial arms at 48 weeks (**A**, left panel), between AZM arm at baseline and 48 weeks (**A**, middle panel) and between AZM arm at 48 and 72 weeks (**A**, right panel). Associations were considered significant using an ANCOM detection level ≥ 0.6. Stars on the right of each bar indicate ANCOM detection levels: levels > 0.6 are presented by 1 star, > 0.7 by 2 stars, > 0.8 by 3 stars and > 0.9 by 4 stars. The raw W statistic for each comparison is shown left of the stars. The horizontal length of each bar indicates the mean centered log ratio (CLR) difference in relative abundance. **B** Boxplots of log_10_ relative abundances of bacterial genera detected as differentially abundant by ANCOM2 by trial arm at 48 weeks (**B**) and within AZM arm at baseline, 48 and 72 weeks (**C**)

To detect which genera were driving the changes in beta diversity observed between the trial arms at 48 weeks, we tested for differentially abundant taxa using ANCOM2. At baseline, no taxon was differentially abundant between the trial arms. At 48 weeks, 13 genera were differentially abundant between trial arms (Fig. 5 and S21). *Moraxella*, *Haemophilus*, *Aggregatibacter*, *Streptobacillus*, *Peptococcus*, ASV 205 (*Clostridia*_UCG-014) and ASV-62 (*Lachnospiraceae*) had significantly lower relative abundance in the AZM arm compared to placebo (Fig. 5). In contrast, *Veillonella*, *Rothia*, *Lautropia*, *Actinomyces*, *Treponema* and *Oribacterium* had increased in relative abundance in the AZM arm (Fig. 5A). Fifteen genera were detected to be differentially abundant in the samples from participants in the AZM arm between baseline and 48 weeks (Fig. 5A). There was a high degree of concordance between those genera detected as differentially abundant between AZM and placebo arms at 48 weeks and those detected as differentially abundant between the baseline and 48 weeks samples in the AZM arm (12 out of 16 genera were concordant, all with the same direction of effect) Fig. 5A.

When comparing baseline with 72 weeks, many of the same genera (*Lautropia*, *Treponema*, *Rothia*, *Peptococcus*, ASV_209 *Clostridia*, ASV_157 *Absconditabacteria*, *Aggregatibacter*, *Moraxella*, *Haemophilus*) that were differentially abundant when comparing baseline with 48 weeks remained differentially abundant, with the same direction of effect, however the magnitude of the difference (estimated by CLR mean difference) was generally reduced (Fig. 5A). Interestingly, several genera (*Veillonella*, *Oribacterium*, *Streptobacillus*, ASV_205 *Clostridia*, ASV_62 Lachnospiraceae) showed opposite directions of effect when comparing baseline to 48 weeks with baseline to 72 weeks.

### Concordance between differential abundance testing methods

We used other statistical methods for comparison with the differential abundance results from ANCOM2, which generally showed strong concordance, supplementary material (Figure S24, Supplementary Table S5-S11). At 48 weeks, five genera were detected as differentially abundant between study arms by all methods (*Lautropia*, *Moraxella*, *Rothia*, *Treponema* and *Veilonella)* (Figure S24). *Lautropia*, *Moraxella*, *Treponema*, *Oribacterium*, *F0058* and *ASV 209* were detected as differentially abundant between the baseline and 48-week samples from participants in the AZM arm by all methods (Figure S25). *Moraxella* was the only genus detected as differentially abundant between the 48- and 72-week samples from participants in the AZM arm by all methods (Figure S26). There was also a high level of agreement between the differential abundance testing and the SIMPER analysis of the contribution of taxa to overall dissimilarity (Bray Curtis distance) Table S12.

### Associations between relative abundance of selected bacteria genera and clinical measures

We used a linear mixed effect model to identify possible associations between lung function (FEV1z, FVCz) and bacterial genera. Relative abundance of *Neisseria* was positively associated with FEV1z (coefficient, 2.85, standard error, 0.7, *q* = 0.01) whilst that of *Haemophilus* was negatively associated (coefficient, − 6.1, standard error, 1.2, *q* < 0.001). In a separate model each, the adverse events of hospitalisation, additional antibiotic use and acute respiratory exacerbation during intervention were not associated with any of the five bacterial genera previously identified as associated with HCLD (*Streptococcus*, *Prevotella*, *Haemophilus*, *Moraxella* and *Neisseria*) [17].

We next used a univariate linear regression effect model to study whether change in lung function between visits over the study period was associated with changes in the relative abundances of the five bacterial genera of interest or with change in within-participant beta diversity over time. In the AZM arm, within-participant increase in FEV1*z* was positively associated with within-participant increase in the relative abundance of *Streptococcus* (coefficient [standard error], 3.2 [1.11], *q* = 0.01, Table 3). In contrast, an increase in FEV1*z* was negatively associated with within-participant increase in the relative abundance of *Moraxella* (− 2.74 [0.74], *q* = 0.002, Table 3). Furthermore, in the AZM arm, an increase in within-participant Aitchison distance was positively associated with within-participant increase in both FEV1*z* (1.05 [0.45], *p* = 0.02, Table S13) and FVC*z *(0.95 [0.42], *p* = 0.02 Table S13).

**Table 3:** Univariate linear regression analysis of within-participant change in FEV1*z* and FVC*z* and within-participant change in relative abundance of selected genera (outcome) between visits

Within-participant change in genus	Trial arm	Within-participant change in FEV1z				Within-participant change in FVCz			
		coef	stderr	pval	qval	coef	stderr	pval	qval
*Streptococcus*	AZM	3.22	1.11	**0.004**	**0.01**	2.66	1.04	**0.01**	0.08
	Placebo	− 0.79	1.46	0.59	0.82	− 0.42	1.2	0.72	0.93
*Prevotella*	AZM	0.1	0.86	0.91	0.97	0.59	0.78	0.45	0.63
	Placebo	− 1.54	1.08	0.16	0.82	− 1.56	0.87	0.07	0.51
*Haemophilus*	AZM	− 0.92	1.95	0.64	0.89	− 1.12	1.82	0.54	0.63
	Placebo	− 0.76	2.86	0.79	0.9	0.15	2.3	0.95	0.95
*Moraxella*	AZM	− 2.74	0.74	**0.0003**	**0.002**	− 1.28	0.7	0.07	0.25
	Placebo	0.18	1.42	0.9	0.9	0.92	1.11	0.41	0.93
*Neisseria*	AZM	0.05	1.43	0.97	0.97	− 0.34	1.33	0.8	0.8
	Placebo	1.5	1.83	0.41	0.82	2.66	1.04	**0.01**	0.08

Associations were tested with MaAsLin2 using a linear regression model with FEV1z or FVCz and trial arm as fixed effects and within-participant change in relative abundance of selected genera as outcome. Statistical significance was corrected for multiple testing using Benjamini/Hochberg correction. Columns correspond to the within-participant change in genus, trial arm, the coefficient estimate (coef) and standard error from the model (stderr), nominal *p*-value (pval), and FDR corrected *p*-value (qval). Number of samples in azithromycin (AZM) and placebo arms are 377 and 365 respectively

## Discussion

We investigated the impact of long-term AZM treatment on the diversity and composition of the sputum bacteriome of children and adolescents with HCLD and the persistence of this effect six months post-intervention. AZM treatment was associated with reduced bacterial load and maintenance of within-sample (Shannon) diversity compared with placebo. In turn, low bacterial load and high Shannon alpha diversity were associated with better lung function measures. Treatment with AZM was associated with a reduction in the relative abundance of several potentially pathogenic taxa at 48 weeks, including *Haemophilus* and *Moraxella*. These changes persisted, but were less marked, at 72 weeks. The relative abundance of *Haemophilus* was negatively associated with lung function (FEV1*z*), whilst an increase in the relative abundance of *Moraxella* over time was associated with a decline in lung function. In contrast, the bacterial genera *Neisseria* and *Streptococcus* were associated with improved lung function measures.

The reduction in the total sputum bacterial load after 48 weeks of AZM treatment is expected, given the antibacterial activity and immune modulatory effects of AZM [15, 47] and may have contributed to the reduction in the frequency of acute respiratory exacerbations in the AZM arm [7]. The difference in the bacterial load between the arms at 48 weeks represents an approximately 3-fold reduction in bacterial 16S rRNA gene copies and may be biologically relevant. Sputum bacterial load returned to baseline levels by 72 weeks. Interestingly, other studies of long-term administration of AZM did not observe any effect on total bacterial burden measured by 16 rRNA gene copy numbers [11, 12, 48] or quantitative culture [48]. The reasons for the inconsistent findings may include differences in dosage, duration and frequency of AZM treatment, varying samples types (bronchoalveolar lavage vs sputum), HIV-status, prior antibiotic exposure, smoking status, age and variations between different CLDs (COPD [11, 48], asthma [12], cystic fibrosis [10]). For instance, AZM would likely have less effect on bacterial load if the dominant taxa, e.g. *Pseudomonas* or *Staphylococcus spp*. (in cystic fibrosis), are resistant to AZM.

Low alpha diversity is a signal for dysbiotic bacterial communities in many diseases [49, 50]. We observed a decline in Shannon alpha diversity in the placebo, but not the AZM arm, over time. High alpha diversity (Shannon index) was associated with enhanced lung function (FEV1*z* and FVC*z*). Our findings are consistent with those of Rogers et al. [51] who showed that lower Shannon alpha diversity of the sputum bacteriome was associated with lower FEV1*z* in adult participants with non-cystic fibrosis bronchiectasis treated with twice-daily erythromycin for 12 months.

In our study, low Shannon alpha diversity was also associated with the development of adverse events in the placebo arm. The decrease in Shannon alpha diversity observed in the placebo arm at 48 weeks may, in part, have been a consequence of the additional antibiotic exposure due to the more frequent acute respiratory exacerbations and hospitalisations occurring in this arm. This additional antibiotic exposure may also explain our observation that the median within-participant change in the bacteriome profiles of placebo arm participants who experienced adverse events during the trial was greater than those that did not. Other studies have described changes in microbial profiles associated with treatment of acute exacerbations of chronic obstructive pulmonary disease [19].

Interestingly, AZM did not affect Shannon alpha diversity (which is a measure of both species richness and evenness) of the sputum bacteriome at any visit. This observation is consistent with the findings of Acosta et al. [10] in which 2 years of AZM treatment did not affect the Shannon alpha diversity of cystic fibrosis sputum bacteriome [10] but contrasts with the findings of Segal *et al*. [11] who observed a reduction in Shannon alpha diversity in emphysema patients treated with AZM.

Bray-Curtis within-group dissimilarity was lower than between-group dissimilarity, when comparing the 48-week samples in the AZM arm with baseline samples in the AZM arm or the 48-week samples in the placebo arm. Our findings are consistent with previous studies in asthma [12], COPD [11] and bronchiolitis [52] participants. In contrast, Acosta [10] did not observe any effect of AZM on the beta diversity of the sputum bacteriome of cystic fibrosis participants. This difference in findings may be explained by the more prolonged duration of treatment—2 years compared to 48 weeks or less in the other studies—over which period the effect of AZM may diminish due to macrolide resistance or poor drug adherence. In our study, beta diversity (within-participant Aitchison distance) in the AZM arm between study visits was also associated with improvement in lung function measures over the same period, suggesting that the AZM-mediated change in composition of the sputum bacteriome may contribute to improved lung function in this population.

We identified 13 differentially abundant taxa including *Moraxella*, *Haemophilus* (reduced) and *Rothia*, *Veillonella*, *Treponema* and *Lautropia* (enriched) in the AZM compared with placebo arms at 48 weeks*.* The high degree of concordance (12/13) between these taxa and those identified as differentially abundant between the baseline and 48-week samples in the AZM arm indicates that this finding is unlikely to represent false discovery. Many of the same taxa were identified by multiple different methods for differential abundance testing. These data are supported by our previous analysis of sputum bacterial culture from this cohort, which showed a reduction in the prevalence of *Moraxella catarrhalis* and *Haemophilus influenzae* at 48 weeks [53] compared with baseline.

In a separate cross-sectional study [17], we identified increased risk for HCLD in children with sputum bacteriome dominated by *Haemophilus*, *Moraxella* or *Neisseria* (HMN) compared with bacteriome dominated by *Prevotella* or *Streptococcus*. We also showed that cell-free products of HMN sputum bacteriome induced epithelial disruption and inflammatory responses in vitro [17]. Our findings from the present study support the central role of *Haemophilus* and *Moraxella* in HCLD. Improvement in FEV1*z* was negatively associated with *Moraxella* relative abundance. Interestingly, the reduced relative abundance of *Haemophilus* and *Moraxella* at 48 weeks in the AZM arm persisted, but with reduced magnitude, at 72 weeks. Further work is required to explore the persistent effect of AZM on these taxa beyond 72 weeks and associated changes in lung function. The relative abundance of *Streptococcus* was positively associated with FEV1*z*, which is consistent with our earlier finding study [17] that this genus may contain species that may be protective in HCLD. Given that streptococcal species vary widely in pathogenic potential, species-specific discrimination is likely to be critical. Therefore, further research is needed to unravel if and which members of the *Streptococcus* genus are associated with a protective effect. Also, it is also possible that the association between the relative abundance of *Streptococcus* genus and FEV1*z* may be due to removal of pathogenic species and/or inflammatory insult that encourages proliferation of the genus as a bystander effect. Interestingly, in the current study, the relative abundance of *Neisseria* was positively associated with FEV1*z*, in contrast to our previous findings. Again, species-specific discrimination may resolve these discrepant findings.

Our study has several strengths. It is the first study to assess the effect of long-term AZM treatment on the sputum bacteriome of African children with HCLD. The double-blinded, randomised, placebo-controlled design allows more direct inference of causal relationships. It is also the first study to analyse the persistence of AZM effect on the sputum bacteriome six months after treatment cessation using this study design. Our study is limited by several factors. 16S rRNA amplicon sequencing is unable to resolve taxonomy to the species level, which may be important, for example given the widely varying pathogenicity of *Streptococcus* species. The use of sputum to assess the lower airway microbiota is limited by contamination from the upper airways. However, previous studies have demonstrated acceptable concordance between the sputum and bronchoalveolar lavage bacteriome, [54, 55] in asthma [55] and cystic fibrosis participants [54]. The 16S rRNA gene qPCR that we used is subject to bias due to varying copy number of 16S rRNA genes in bacteria.

## Conclusion

In conclusion, 48 weeks of once-weekly AZM reduced the total bacterial load and preserved within-sample bacterial diversity of children and adolescents with HCLD, features that were associated with better lung function measures. Our results confirm and extend previous findings, from a separate cohort, that *Haemophilus* and *Moraxella* likely play a central role in the pathogenesis of HCLD whilst *Streptococcus* may include species with a protective effect. Modulation of the airway microbiota, with a targeted reduction in disease-associated taxa, may be a strategy to ameliorate disease in people with HCLD.

## Supplementary Information

**Additional file 1: Figure S1.** A bar plot of the taxa and their relative abundance of the extraction and sequencing mock controls compared to manufacturer profiles. **Figure S2.** A scatterplot showing the correlation between samples repeated within a run (WR, n = 74) and between runs (BR, n=28). **Figure S3.** A scatterplot showing the spread of biological samples (n=960) and the negative controls (primestore, n=43) 16S copies vs final number of reads (A1 and A2), Shannon alpha diversity index (B1 and B2) and age of participant in years (C1 and C2). **Figure S4.** Ordination plots of showing the spread of biological samples (n=960) and the negative controls (primestore, n=43) coloured by their 16S copies. **Figure S5.** Ordination plots showing the spread of biological samples (n=960) and the negative controls (primestore, n=43) coloured by their number of reads. **Figure S6.** Ordination plots showing the spread of biological samples (n=960) and the negative controls (primestore, n=43) coloured by the age of the participant. **Figure S7.** Rarefaction curves showing number of ASVs detected and 16S copies of samples. **Figure S8.** Rarefaction curves showing number of ASVs detected and number of reads of samples. **Figure S9.** Bar plot showing the profiles of biological samples with <100 16S copies (n=2) in comparison to Primestores profiles (n=43). **Figure S10.** Bar plot showing the profiles of biological samples with >100 to <1000 16S copies (n=10) in comparison to Primestores profiles (n=43). **Figure S11.** Ordination plots showing the profiles of a subset of biological samples with low 16S copies and the negative controls. **Figure S12.** Ordination plots showing the profiles of a subset of biological samples with low reads and the negative controls. **Figure S13.** Ordination plots showing the spread of biological samples (n=960) and the negative controls (primestore, n=43) coloured by the run in which the sample was processed. **Figure S14.** Ordination plots showing the spread of biological samples (n=960) and the negative controls (primestore, n=43) coloured by the country of sampling. **Figure S15.** Ordination plots showing the spread of biological samples (n=960) and the negative controls (primestore, n=43) coloured by visit. **Figure S16.** Ordination plots showing the spread of biological samples (n=960) and the negative controls (primestore, n=43) coloured by the age at sampling. **Figure S17.** Output from decontamination analysis using the DECONTAM R package. **Figure S18.** Boxplot of Shannon alpha diversity index between trial arms at each visit (A) and between study visits in AZM (B) and Placebo (C) arms. **Figure S19.** Violin boxplot comparing two beta diversity metrics between samples collected from participants in the AZM arms at baseline and 48 weeks, 48 and 72 weeks and baseline and 72 weeks. **Figure S20.** Violin boxplot comparing two beta diversity metrics between samples collected from participants in the Placebo arms at baseline and 48 weeks, 48 and 72 weeks and baseline and 72 weeks. **Figure S21.** Principal Coordinates Analysis of Atchison (A) and Bray-Curtis (B) [on unrarefied ASV counts] distance matrixes between trial arms at each visit. **Figure S22.** Barplot of the relative abundances of the top 10 most prevalent phyla in all samples. **Figure S23.** Barplot of the relative abundances of the top 12 most prevalent genera in all samples. **Figure S24.** Heatmap displaying the q values of the genera detected as differentially abundant between AZM and placebo arms at 48 weeks by 10 statistical methods. **Figure S25.** Heatmap displaying the q values of the genera detected as differentially abundant within the AZM arm between baseline and 48-week samples by 10 methods. **Figure S26.** Heatmap displaying the q values of the genera detected as differentially abundant within the AZM arm between 48- and 72-week samples by 10 methods. **Table S1.** The taxonomy of the ASVs in the extraction and sequencing control. **Table S2.** List of 70 ASVs detected by the DECONTAM R package as potential contaminants based on comparison between biological samples and negative controls. **Table S3.** The association between bacterial load (16S rRNA copies) and selected variables using linear mixed effects modelling. **Table S4.** The association between Shannon diversity indices and selected variables using linear mixed effects modelling. **Table S5.** Results of differential abundance testing of bacterial taxa from AZM and Placebo samples from 48 weeks using 10 methods. **Table S6.** Results of differential abundance testing of bacterial taxa from AZM and Placebo samples from 72 weeks using DESeq2. **Table S7.** Results of differential abundance testing of bacterial taxa from AZM and Placebo samples from 72 weeks using Ancom-II. **Table S8.** Results of differential abundance testing of bacterial taxa from samples from the AZM arm at baseline and 48 72 weeks using 10 methods. **Table S9.** Results of differential abundance testing of bacterial taxa from samples from the AZM arm at 48 and 72 weeks using 10 methods. **Table S10.** Results of differential abundance testing of bacterial taxa from Placebo samples from 48 and 72 weeks using DESeq2. **Table S11.** Results of differential abundance testing of bacterial taxa from Placebo samples from baseline and 72 weeks using DESeq2. **Table S12.** Contributions of top genera to overall dissimilarity between AZM and Placebo arms at 48 weeks and, within the AZM arm, between Baseline and 48-week samples- SIMPER analysis. **Table S13.** Univariate linear regression analysis of within-participant Aitchison distance (outcome) and within-participant change in lung function metrics (FVCz and FEV1z) between visits.

## Data Availability

The 16S rRNA amplicon sequences, anonymised clinical, laboratory and socio-demographic data and analysis codes that support the findings of this study are available in Sequence Read Archive (accession number PRJNA769290) and GitHub repository (https://github.com/ReginaEsinamAbotsi/BREATHE_Sputum_bacteriome_analysis).
