# Supplementary material for: Sputum bacterial load and bacterial composition correlate with lung function and are altered by long-term azithromycin treatment in children with HIV-associated chronic lung disease
Source: Microbiome. 2023 Feb 20;11:29. doi: 10.1186/s40168-023-01460-x (PMC9940396; doi:10.1186/s40168-023-01460-x)
Supplement: Supplementary file 2 — Additional file 1: Figure S1. A bar plot of the taxa and their relative abundance of the extraction and sequencing mock controls compared to manufacturer profiles. Figure S2. A scatterplot showing the correlation between samples repeated within a run (WR, n = 74) and between runs (BR, n=28). Figure S3. A scatterplot showing the spread of biological samples (n=960) and the negative controls (primestore, n=43) 16S copies vs final number of reads (A1 and A2), Shannon alpha diversity index (B1 and B2) and age of participant in years (C1 and C2). Figure S4. Ordination plots of showing the spread of biological samples (n=960) and the negative controls (primestore, n=43) coloured by their 16S copies. Figure S5. Ordination plots showing the spread of biological samples (n=960) and the negative controls (primestore, n=43) coloured by their number of reads. Figure S6. Ordination plots showing the spread of biological samples (n=960) and the negative controls (primestore, n=43) coloured by the age of the participant. Figure S7. Rarefaction curves showing number of ASVs detected and 16S copies of samples. Figure S8. Rarefaction curves showing number of ASVs detected and number of reads of samples. Figure S9. Bar plot showing the profiles of biological samples with <100 16S copies (n=2) in comparison to Primestores profiles (n=43). Figure S10. Bar plot showing the profiles of biological samples with >100 to <1000 16S copies (n=10) in comparison to Primestores profiles (n=43). Figure S11. Ordination plots showing the profiles of a subset of biological samples with low 16S copies and the negative controls. Figure S12. Ordination plots showing the profiles of a subset of biological samples with low reads and the negative controls. Figure S13. Ordination plots showing the spread of biological samples (n=960) and the negative controls (primestore, n=43) coloured by the run in which the sample was processed. Figure S14. Ordination plots showing the spread of biological samples (n=960) [file 40168_2023_1460_MOESM1_ESM.zip › Supplementary_material_Abotsi_19012023_FINAL.docx]

**Sputum bacterial load and bacterial composition correlate with lung function and are altered by long term azithromycin treatment in children with HIV-associated chronic lung disease– Supplementary materials**

Regina E. Abotsi^1,2*^, Felix S. Dube^1^, Andrea M. Rehman^3^, Shantelle Claassen-Weitz^4^, Yao Xia^5^, Victoria Simms^3,6^, Kilaza S. Mwaikono^7,8^, Sugnet Gardner-Lubbe^9^, Grace McHugh^6^, Lucky G. Ngwira^10,11^, Brenda Kwambana-Adams^12^, Robert S Heyderman^12^, Jon Ø Odland^13,14,15^, Rashida A Ferrand^6,16^, Mark P. Nicol^4,5^ and The BREATHE study team

1. Department of Molecular and Cell Biology & Institute of Infectious Diseases and Molecular Medicine, University of Cape Town, Cape Town, South Africa
2. Department of Pharmaceutical Microbiology, School of Pharmacy, University of Health and Allied Sciences, Ho, Ghana
3. International Statistics and Epidemiology Group, London School of Hygiene and Tropical Medicine, London, United Kingdom
4. Division of Medical Microbiology, Department of Pathology, University of Cape Town, Cape Town, South Africa
5. Division of Infection and Immunity, School of Biomedical Sciences, University of Western Australia, Perth, Australia
6. Biomedical Research and Training Institute, Harare, Zimbabwe
7. Computational Biology Group and H3ABioNet, Department of Integrative Biomedical Sciences, University of Cape Town, Cape Town, South Africa.
8. Department of Science and Laboratory Technology, Dar es Salaam Institute of Technology, Dar es Salaam, Tanzania
9. Department of Statistics and Actuarial Science, Stellenbosch University, Stellenbosch, South Africa.
10. Malawi-Liverpool Wellcome Trust Clinical Research Programme, Blantyre, Malawi.
11. Department of Clinical Sciences, Liverpool School of Tropical Medicine, Liverpool, United Kingdom
12. NIHR Global Health Research Unit on Mucosal Pathogens, Research Department of Infection, Division of Infection and Immunity, University College London, London, United Kingdom
13. Department of Community Medicine, University of Tromsø, Tromsø, Norway
14. International Research Laboratory for Reproductive Ecotoxicology (IL RET), The National Research University Higher School of Economics, Moscow, Russia
15. School of Health Systems and Public Health, University of Pretoria, Pretoria, South Africa
16. Clinical Research Department, London School of Hygiene and Tropical Medicine, London, United Kingdom

*****Corresponding Author: Prof Mark P. Nicol

Tel: +61 8 6457 6964

E-mail address: Mark.Nicol@uwa.edu.au

Postal address: The University of Western Australia, M504, Perth WA 6009 Australia

# TABLE OF CONTENT

[TABLE OF CONTENT	2](#_Toc122330589)

[LIST OF FIGURES	3](#_Toc122330590)

[LIST OF TABLES	5](#_Toc122330591)

[SECTION 1. DETAILED DESCRIPTION OF METHODS	6](#_Toc122330592)

[1.1.1.	Differentially abundant taxa and SIMPER analysis.	6](#_Toc122330593)

[a.	Mann–Whitney–Wilcoxon test on Total sum scaled ASV counts	6](#_Toc122330594)

[b.	Mann–Whitney–Wilcoxon test on variance stabilising transformed (VST) ASV counts	6](#_Toc122330595)

[c.	Mann–Whitney–Wilcoxon test on centre log ratio transformed (CLR) ASV counts	6](#_Toc122330596)

[d.	DESeq2	7](#_Toc122330597)

[e.	Analysis of compositions of microbiomes with bias correction (ANCOM‐BC)	7](#_Toc122330598)

[f.	Aldex2	7](#_Toc122330599)

[g.	Corncob	7](#_Toc122330600)

[h.	MaAsLin 2 - Normalisation = TSS, transformation= LOG, fixed effects= trial_arm	7](#_Toc122330601)

[i.	MaAsLin 2 Normalisation = NONE, transformation= NONE, fixed effects= trial_arm	7](#_Toc122330602)

[j.	ANCOM-II	8](#_Toc122330603)

[SECTION 2. SUPPLEMENTARY RESULTS	8](#_Toc122330604)

[2.1. Results of Quality Control Steps	8](#_Toc122330605)

[2.1.1. Introduction	8](#_Toc122330606)

[a.	Extraction and Sequencing Controls	8](#_Toc122330607)

[b.	Reproducibility within and between the three runs	9](#_Toc122330608)

[c.	Relationship between the biological samples and negative controls (Primestore) profiles	10](#_Toc122330609)

[d.	*In silico* correction of contamination and spurious ASVs	19](#_Toc122330610)

[e.	Alpha diversity	27](#_Toc122330611)

[f.	Beta diversity- Azithromycin only	28](#_Toc122330612)

[g.	Beta diversity- Placebo only	29](#_Toc122330613)

[h.	Beta diversity- Azithromycin and Placebo	30](#_Toc122330614)

[i.	Relative abundance of Phyla in all samples	31](#_Toc122330615)

[j.	Relative abundance of Genera in all samples	32](#_Toc122330616)

[2.2. Results of differential abundance of taxa testing	33](#_Toc122330617)

[2.2.1. AZM and Placebo	33](#_Toc122330618)

[a.	AZM and Placebo at baseline	33](#_Toc122330619)

[b.	AZM and Placebo at 48 weeks	33](#_Toc122330620)

[c.	AZM and Placebo at 72 weeks.	33](#_Toc122330621)

[2.2.2. Azithromycin arm only	34](#_Toc122330622)

[a.	AZM at baseline and 48 weeks	34](#_Toc122330623)

[b.	AZM at 48 and 72 weeks	34](#_Toc122330624)

[c.	AZM at baseline and 72 weeks	35](#_Toc122330625)

[2.2.3. Placebo arm only	35](#_Toc122330626)

[a.	Placebo at baseline and 48 weeks	35](#_Toc122330627)

[b.	Placebo at 48 and 72 weeks	35](#_Toc122330628)

[c.	Placebo at baseline and 72 weeks	35](#_Toc122330629)

[2.3. Results of SIMPER analysis	37](#_Toc122330630)

[2.4. Results of linear regression of within-participant change in beta diversity and lung function.	38](#_Toc122330631)

[REFERENCES	38](#_Toc122330632)

# LIST OF FIGURES

[Figure S 1. A bar plot of the taxa and their relative abundance of the extraction and sequencing mock controls compared to manufacturer profiles.	9](#_Toc122329649)

[Figure S 2. A scatterplot showing the correlation between samples repeated within a run (WR, n = 74) and between runs (BR, n=28).	10](#_Toc122329650)

[Figure S 3. A scatterplot showing the spread of biological samples (n=960) and the negative controls (primestore, n=43) 16S copies vs final number of reads (A1 and A2), Shannon alpha diversity index (B1 and B2) and age of participant in years (C1 and C2).	11](#_Toc122329651)

[Figure S 4. Ordination plots of showing the spread of biological samples (n=960) and the negative controls (primestore, n=43) coloured by their 16S copies.	12](#_Toc122329652)

[Figure S 5.Ordination plots showing the spread of biological samples (n=960) and the negative controls (primestore, n=43) coloured by their number of reads.	12](#_Toc122329653)

[Figure S 6. Ordination plots showing the spread of biological samples (n=960) and the negative controls (primestore, n=43) coloured by the age of the participant.	13](#_Toc122329654)

[Figure S 7. Rarefaction curves showing number of ASVs detected and 16S copies of samples.	13](#_Toc122329655)

[Figure S 8. Rarefaction curves showing number of ASVs detected and number of reads of samples.	14](#_Toc122329656)

[Figure S 9. Bar plot showing the profiles of biological samples with <100 16S copies (n=2) in comparison to Primestores profiles (n=43).	14](#_Toc122329657)

[Figure S 10. Bar plot showing the profiles of biological samples with >100 to <1000 16S copies (n=10) in comparison to Primestores profiles (n=43).	15](#_Toc122329658)

[Figure S 11. Ordination plots showing the profiles of a subset of biological samples with low 16S copies and the negative controls.	15](#_Toc122329659)

[Figure S 12. Ordination plots showing the profiles of a subset of biological samples with low reads and the negative controls.	16](#_Toc122329660)

[Figure S 13. Ordination plots showing the spread of biological samples (n=960) and the negative controls (primestore, n=43) coloured by the run in which the sample was processed.	17](#_Toc122329661)

[Figure S 14. Ordination plots showing the spread of biological samples (n=960) and the negative controls (primestore, n=43) coloured by the country of sampling.	17](#_Toc122329662)

[Figure S 15. Ordination plots showing the spread of biological samples (n=960) and the negative controls (primestore, n=43) coloured by visit.	18](#_Toc122329663)

[Figure S 16. Ordination plots showing the spread of biological samples (n=960) and the negative controls (primestore, n=43) coloured by the age at sampling.	18](#_Toc122329664)

[Figure S 17. Output from decontamination analysis using the DECONTAM R package.	19](#_Toc122329665)

[Figure S 18. Boxplot of Shannon alpha diversity index between trial arms at each visit (A) and between study visits in AZM (B) and Placebo (C) arms.	27](#_Toc122329666)

[Figure S 19. Violin boxplot comparing two beta diversity metrics between samples collected from participants in the AZM arms at baseline and 48 weeks, 48 and 72 weeks and baseline and 72 weeks.	28](#_Toc122329667)

[Figure S 20. Violin boxplot comparing two beta diversity metrics between samples collected from participants in the Placebo arms at baseline and 48 weeks, 48 and 72 weeks and baseline and 72 weeks.	29](#_Toc122329668)

[Figure S 21. Principal Coordinates Analysis of Atchison (A) and Bray-Curtis (B) [on unrarefied ASV counts] distance matrixes between trial arms at each visit.	30](#_Toc122329669)

[Figure S 22. Barplot of the relative abundances of the top 10 most prevalent phyla in all samples.	31](#_Toc122329670)

[Figure S 23. Barplot of the relative abundances of the top 12 most prevalent genera in all samples.	32](#_Toc122329671)

[Figure S 24. Heatmap displaying the q values of the genera detected as differentially abundant between AZM and placebo arms at 48 weeks by 10 statistical methods.	33](#_Toc122329672)

[Figure S 25. Heatmap displaying the q values of the genera detected as differentially abundant within the AZM arm between baseline and 48-week samples by 10 methods.	34](#_Toc122329673)

[Figure S 26. Heatmap displaying the q values of the genera detected as differentially abundant within the AZM arm between 48- and 72-week samples by 10 methods.	35](#_Toc122329674)

# LIST OF TABLES

[Table S 1. The taxonomy of the ASVs in the extraction and sequencing control.	9](#_Toc122330145)

[Table S 2. List of 70 ASVs detected by the DECONTAM R package as potential contaminants based on comparison between biological samples and negative controls.	20](#_Toc122330146)

[Table S 3. The association between bacterial load (16S rRNA copies) and selected variables using linear mixed effects modelling.	21](#_Toc122330147)

[Table S 4. The association between Shannon diversity indices and selected variables using linear mixed effects modelling.	24](#_Toc122330148)

[Table S 5. Results of differential abundance testing of bacterial taxa from AZM and Placebo samples from 48 weeks using 10 methods.	33](#_Toc122330149)

[Table S 6. Results of differential abundance testing of bacterial taxa from AZM and Placebo samples from 72 weeks using DESeq2.	34](#_Toc122330150)

[Table S 7. Results of differential abundance testing of bacterial taxa from AZM and Placebo samples from 72 weeks using Ancom-II	34](#_Toc122330151)

[Table S 8. Results of differential abundance testing of bacterial taxa from samples from the AZM arm at baseline and 48 72 weeks using 10 methods.	34](#_Toc122330152)

[Table S 9. Results of differential abundance testing of bacterial taxa from samples from the AZM arm at 48 and 72 weeks using 10 methods.	35](#_Toc122330153)

[Table S 10. Results of differential abundance testing of bacterial taxa from Placebo samples from 48 and 72 weeks using DESeq2.	35](#_Toc122330154)

[Table S 11. Results of differential abundance testing of bacterial taxa from Placebo samples from baseline and 72 weeks using DESeq2.	35](#_Toc122330155)

[Table S 12. Contributions of top genera to overall dissimilarity between AZM and Placebo arms at 48 weeks and, within the AZM arm, between Baseline and 48-week samples- SIMPER analysis.	37](#_Toc122330156)

[Table S 13. Univariate linear regression analysis of within-participant Aitchison distance (outcome) and within-participant change in lung function metrics (FVCz and FEV1z) between visits.	38](#_Toc122330157)

# SECTION 1. DETAILED DESCRIPTION OF METHODS

## 1.1.1. Differentially abundant taxa and SIMPER analysis.

Differentially abundant taxa were identified using ANCOM2[1] on unrarefied data with False Discovery Rate (FDR) Benjamini/Hochberg correction (cut off = 0.05). Nine other methods for detecting differentially abundant taxa were used for comparison and reported below (Wilcoxon signed-rank test on data after the following normalisation methods–total sum scaling, variance stabilising transformation, and centred log transformation after applying a pseudo count of one, Aldex2[2], Deseq2[3], Ancombc[4], Corncob[5], MaasLin2[6] with total sum scaling and log transformation, MaasLin2[6] with centred log transformation after applying a pseudo count of one. The relative contribution of each taxon to overall dissimilarity was measured using SIMPER analysis on the Bray-Curtis distances between samples. The input for all the methods is a PHYLOSEQ object merged at the genus level and 0.5% prevalence filtered. No rarefication was applied to the feature tables used in the analysis below. In the first three methods, we applied Wilcoxon test on ASV counts normalised using three different methods to determine how different normalisations will affect the number of genera found to be differentially abundant.

### a. Mann–Whitney–Wilcoxon test on Total sum scaled ASV counts

The ASV counts, previously merged at genus level and 0.5% prevalence filtered, were converted to relative abundances using total sum scaling equation – x/sum(x), where x is ASV counts implemented using the *transform_sample_counts* function embedded in the PHYLOSEQ package. The resulting relative abundances of each genus of AZM and Placebo arms were then compared using the Wilcoxon test. Comparison of samples from different visit but within the same trial arm were done by Wilcoxon test for paired samples. The p values were then adjusted for multiple testing using Benjamin-Hochberg method to produce the q values. The false discovery rate was set to 0.05 hence only genera with *q* values < 0.05 were deemed differentially abundant.

### b. Mann–Whitney–Wilcoxon test on variance stabilising transformed (VST) ASV counts

A pseudocount of one was applied to all ASVs before conversion from PHYLOSEQ to Deseq2 object. This was necessary so that log geometric means can be calculated when the *estimateSizeFactors* function embedded in Deseq2 is later applied. The PHYLOSEQ object was then converted to a Deseq2 object using the *phyloseq_to_deseq2* function so that VST normalisation can be conducted using a function from this package. Size factors (*estimateSizeFactors*) and dispersions (*estimateDispersions*) were estimated before variance stabilising transformation was applied to the data (*getVarianceStabilizedData*). The resulting variance stabilising transformed (VST) ASVs count of each genus of AZM and Placebo arms were then compared using the Wilcoxon test. Comparison of samples from different visit but within the same trial arm were done by Wilcoxon test for paired samples. The p values were then adjusted for multiple testing using Benjamin-Hochberg method to produce the *q* values. The false discovery rate was set to 0.05 hence only genera with *q* values < 0.05 were deemed differentially abundant.

### c. Mann–Whitney–Wilcoxon test on centre log ratio transformed (CLR) ASV counts

Here a pseudocount of one was again applied to allow the calculation of log geometric means. The ASV counts were then CLR transformed using the *transform_sample_counts* function embedded in the PHYLOSEQ package with the function *x/exp(mean(log(x))), x* is ASV counts. The resulting log ratios of each ASV within the genera of AZM and Placebo arms were then compared using the Wilcoxon test. Comparison of samples from different visit but within the same trial arm were done by Wilcoxon test for paired samples. The *p* values were then adjusted for multiple testing using Benjamin-Hochberg method to produce the *q* values. The false discovery rate was set to 0.05 hence only genera with *q* values < 0.05 were deemed differentially abundant.

### d. DESeq2

This analysis is based on negative binomial distribution and makes use of VST normalisation. It does not account for the compositional nature of the data. The PHYLOSEQ object was converted to DESeq2 using the function *phyloseq_to_deseq2.* The DEseq function was applied to this object with Wald test, local *fitType* and poscounts option for *sfType*. The *lfcShrink* function was applied with coef = 2 and type = ”*apeglm*”. The p values were then adjusted for multiple testing using Benjamin-Hochberg method to produce the *q* values. The false discovery rate was set to 0.05 hence only genera with *q* values < 0.05 were deemed differentially abundant.

### e. Analysis of compositions of microbiomes with bias correction (ANCOM‐BC)

The *ancombc* function in the ANCOMBC R package v 1.0.5 was applied to the genus-agglomerated and 0.5% prevalence-filtered ASV counts with the following options: *p* value adjustment was Benjamin-Hochberg, library cut =1000, structural zeros= TRUE, neg_lb= TRUE, conserve=TRUE, global=TRUE. All other options were left as default. The false discovery rate was set to 0.05 hence only genera with *q* values < 0.05 were deemed differentially abundant.

### f. Aldex2

A data frame of the genera counts, and corresponding sample metadata were passed to the *aldex* function in ALDEx2 R package v 1.22.0 setting the denom parameter to “iqlr”. All other parameters were set to default. Both Wilcoxon (ALDEx2 Wilcoxon) and t-test (ALDEx2 t-test) were used for testing differences in genera relative abundances between AZM and Placebo or samples from different visits. The false discovery rate was set to 0.05. The function returned Benjamini-Hochberg (BH) FDR-corrected *p* values.

### g. Corncob

The genus-merged and 0.5% prevalence filtered PHYLOSEQ object was passed to the *differentialTest* function of the corncob R package version 0.2.0. We selected Wald test for significance testing and false discovery rate to Benjamini-Hochberg. All other options were set to default. Since the false discovery rate was set to 0.05 hence only genera with *q* values < 0.05 were deemed differentially abundant.

### h. MaAsLin 2 - Normalisation = TSS, transformation= LOG, fixed effects= trial_arm

A data frame of the genera counts, and corresponding sample metadata were passed to the Maaslin2 function in MaAsLin2 R package v 1.4.0 setting the minimum prevalence to 0.0 because 0.5% prevalence filtered was already applied to the counts. Maximum significance was set to 0.05, standardize was set to FALSE and fixed effect was set as trial arm or visit. All other parameters were set to default. Since the false discovery rate was set to 0.05 hence only genera with *q* values < 0.05 were deemed differentially abundant.

### i. MaAsLin 2 Normalisation = NONE, transformation= NONE, fixed effects= trial_arm

A data frame of the genera ASV counts that have previously been centred-log-ratio transformed and corresponding sample metadata were passed to the Maaslin2 function in MaAsLin2 R package v 1.4.0 setting the minimum prevalence to 0.0 because 0.5% prevalence filtered was already applied to the counts. Maximum significance was set to 0.05, standardize was set to FALSE and fixed effect was set trial arm or visit. Normalisation and transformation were set to “None”. All other parameters were set to default. Since the false discovery rate was set to 0.05 hence only genera with *q* values < 0.05 were deemed differentially abundant.

### j. ANCOM-II

The genus-merged, 0.5% prevalence filtered ASV count table was inputted through the ANCOM-II[1] (<https://github.com/FrederickHuangLin/ANCOM> ) *feature_table_pre_process* function which identified outlier and structural zeros. The trial arm or visit was specified as the group and main variable. The resulting feature table was then passed through the ANCOM function and *p*-values were FDR-corrected using the BH method (alpha set at 0.05). W statistics greater than or equal to 60% of the total number of genera tested were considered significant.

# SECTION 2. SUPPLEMENTARY RESULTS

# 2.1. Results of Quality Control Steps

## 2.1.1. Introduction

A total of 1152 samples (78 biological samples belonging to a comparison group, 12 Zymobiomics extraction controls, 12 Zymobiomics sequencing control, 101 biological repeats, 43 non-template (negative or Primestore) controls and 906 biological samples included in the main trial) including controls were processed in three runs of 384 samples each. To ensure that the extraction step and sequencing steps are validated we included Zymobiomics mock community extraction controls (cells) (catalogue no. ZR D6300, Zymo Research Corp., Irvine, CA, United States) and sequencing controls (DNA) (catalogue no. ZR D6305, Zymo Research Corp., Irvine, CA, United States). We also repeated samples within plates and between plates in the same and between runs, to assess reproducibility. This action is to ensure there are no differences in bacteriome profiles introduced by batch effects. Furthermore, we compared profiles from biological samples with non-template control (in this case, Primestore, which was used as a storage medium of the samples) to assess background contaminating profiles. We then assess whether samples with low biomass (low 16S copies) clustered with the negative controls on a log-ratio biplot, suggesting background contamination rather than true biological signal. Also, we assess clustering of samples based on age, run number, study site (country), visit/timepoint, number of reads and 16S copy numbers which may introduce bias in our analysis. Finally, we used the *isContaminant* function within the DECONTAM R package to determine which ASVs are likely to be contaminants based on the Primestore profiles. This section of the supplementary material contains the results of each of these analyses.

### a. Extraction and Sequencing Controls

The Zymobiomics mock community extraction controls (cells) (catalogue no. ZR D6300, Zymo Research Corp., Irvine, CA, United States) and sequencing controls (DNA) (catalogue no. ZR D6305, Zymo Research Corp., Irvine, CA, United States) were comparable to the theoretical compositions provided by the manufacturer (Figure S1, Table S1). However, the DNA profiles were more comparable than the cells highlighting a small bias in the extraction step probably from the lysis step. The mock communities were included on each plate in each run resulting in a total of 12 samples of the extraction controls and 12 of the sequencing controls.

Extraction control Zymobiomics cells

Sequencing control Zymobiomics DNA

Figure S 1. A bar plot of the taxa and their relative abundance of the extraction and sequencing mock controls compared to manufacturer profiles.

Table S 1. The taxonomy of the ASVs in the extraction and sequencing control.

### b. Reproducibility within and between the three runs

A total of 101 biological specimens were repeated within the three runs with one sample repeated thrice. Of these 74 was repeated in the same run (WR) while 28 were repeated between runs (BR). Reproducibility as measured by R^2^ was very high (> 0.9). Only one between run repeat had an R2 value<0.9 (0.69). With regards to reproducibility, no sample was excluded based on age, 16S copies or number of reads as none of these affected reproducibility Figure S2(A, B and C). Out of the 74 WR repeats, 54 were repeated on the same plate to assess intra-plate reproducibility, while 20 specimens were repeated between plates in the same run to assess inter-plate reproducibility within the same run. Reproducibility for all 74 samples was very high (> 0.91) hence no sample was excluded (Figure S2, D, E and F).

A

B

C

D

E

F

Figure S 2. A scatterplot showing the correlation between samples repeated within a run (WR, n = 74) and between runs (BR, n=28). A) shows reproducibility in relation to age of the participants, B) shows reproducibility in relation to16S copy numbers and C) shows reproducibility in relation to number of final reads. The second row of the figure shows samples repeated within the same plate in the same run (WP, n=54) and between plates in the same run (BP, n=20). D) shows reproducibility in relation to age of the participants, E) shows reproducibility in relation to16S copy numbers and F) shows reproducibility in relation to number of final reads.

### c. Relationship between the biological samples and negative controls (Primestore) profiles

We included 43 Primestores (sample storage media) as negative controls across the three runs (Run 1= 13, Run2=16, Run3 = 14). The Primestores samples used were made up of two different batches used in sample storage to better account for batch-to-batch variations in background profiles. The number of biological samples analysed was 960. There was a total of 3219 ASVs that had greater than zero reads. As our previous experiences and that of others[7,8] has shown that age is positively correlated with biomass (16S copies) and this also correlates with the number of reads and sometimes with alpha diversity, we sort to assess this in our data. We found no correlation between the final reads and 16S copies (r=0.05) Figure S3 (A1 and A2) and none between age and 16S copies (r=0.07) Figure S3 (C1 and C2). However, we detected a slight negative correlation between Shannon diversity index and 16S copies (r= -0.26) Figure S3 (B1 and B2).

A2

A1

B2

B1

C2

C1

Figure S 3. A scatterplot showing the spread of biological samples (n=960) and the negative controls (primestore, n=43) 16S copies vs final number of reads (A1 and A2), Shannon alpha diversity index (B1 and B2) and age of participant in years (C1 and C2).

Next, we investigated whether our low biomass specimens (<500 /μl copies, n = 4) shared bacteriome profiles with our negative controls (cluster together on ordination plots). We observed that this was not the case, Figure S4. However, we did excluded specimens with < 100 16S gene copies (n = 2). Our further analysis revealed that samples with low read counts (<1000 reads) may cluster with negative controls indicating similar bacteriome profiles, Figure S5. We therefore excluded these samples as well (read counts were 0 – 4, n = 5). As we and others have shown that these low biomass specimens produced poor reproducible sequencing profiles[7,8]. Four Primestores had <100 16S copies (n = 4) while a large number had >1000 reads (39 of the 43). Specimens collected at younger ages do not seem to cluster with negative controls, Figure S6. We assessed the relationship between the number of ASVs detected and the 16S copies and number of reads. We observed that the number of ASVs detected negatively correlated with low 16S copies (<500), Figure S7 and low number of reads, Figure S8. This observation further supports our exclusion of biological samples with <500 copies and/or <1000 reads.

Figure S 4. Ordination plots of showing the spread of biological samples (n=960) and the negative controls (primestore, n=43) coloured by their 16S copies. Low biomass samples (low 16S copy numbers) did not seem to cluster with negative controls. Only four (4) biological samples had <300 16S copies, the remaining red data points represent Primestores.

Figure S 5.Ordination plots showing the spread of biological samples (n=960) and the negative controls (primestore, n=43) coloured by their number of reads. Low read count samples (<1,000 reads) may cluster with negative controls

Figure S 6. Ordination plots showing the spread of biological samples (n=960) and the negative controls (primestore, n=43) coloured by the age of the participant. Specimens collected at younger ages do not seem to cluster with negative controls.

Figure S 7. Rarefaction curves showing number of ASVs detected and 16S copies of samples. The number of ASVs detected negatively correlated with low 16S copies (<500).

Figure S 8. Rarefaction curves showing number of ASVs detected and number of reads of samples. The number of ASVs detected negatively correlated with low read counts (<1000).

Next, we used a bar plot to visualise the ASV profiles of the two samples with less than 100 16S copies, Figure S9 and the ten samples with >100 to <1000 16S copies, Figure S10 in comparison to the Primestore samples to further assess similarity in profiles. Finally, we constructed an ordination plots (log-ratio biplot, PCA and PCoA) on the subset of biological samples with low 16S copies, Figure S11 and low read counts, Figure S12 to assess whether they will cluster with the Primestore samples.

Figure S 9. Bar plot showing the profiles of biological samples with <100 16S copies (n=2) in comparison to Primestores profiles (n=43).

Figure S 10. Bar plot showing the profiles of biological samples with >100 to <1000 16S copies (n=10) in comparison to Primestores profiles (n=43).

Figure S 11. Ordination plots showing the profiles of a subset of biological samples with low 16S copies and the negative controls. There is a separation between the biological samples with low 16S copies and the negative controls (Primestore, n=43).

Figure S 12. Ordination plots showing the profiles of a subset of biological samples with low reads and the negative controls. There is a separation between the biological samples with low reads and the negative controls (primestore, n=43).

We continue to explore whether our specimens clusters together based on a) which of the three runs, the samples were processed in (run 1, 2 or 3), b) the country of origin of the sample (Zimbabwe or Malawi), c) the sampling time point or visit (baseline, 12 months (48 weeks) and 18 months (72 weeks)), d) sampling method (expectoration vs. induction) and their relationship with negative controls, e) age especially for younger ages (6-10 years and 11 to 19 years). We did not observe any clustering pattern based on run numbers (Figure S13), country (study site) from which the samples were collected (Figure S14) or the visit or timepoint at which the sample was collected, Figure S15. Specimens collected at younger ages do not seem to cluster with negative controls, Figure S16. We detected no clustering based on any of these variables nor with negative controls. Hence no sample was excluded on the basis on any of these criteria.

Figure S 13. Ordination plots showing the spread of biological samples (n=960) and the negative controls (primestore, n=43) coloured by the run in which the sample was processed. No clustering patterns based on run numbers.

Figure S 14. Ordination plots showing the spread of biological samples (n=960) and the negative controls (primestore, n=43) coloured by the country of sampling. No clustering patterns based on country.

Figure S 15. Ordination plots showing the spread of biological samples (n=960) and the negative controls (primestore, n=43) coloured by visit. No clustering patterns based on visit.

Figure S 16. Ordination plots showing the spread of biological samples (n=960) and the negative controls (primestore, n=43) coloured by the age at sampling. No clustering patterns based on age.

### d. *In silico* correction of contamination and spurious ASVs

The number of biological samples remaining is 953 after excluding 7 samples (2<100 copies and 5<1000 reads). The analysis using the DECONTAM R package was conducted on 43 Primestores (negative controls) and 953 biological samples. (Figure S17). The exclusion of the seven biological samples reduced the initial number of ASVs with reads greater than zero from 3219 to 3216. A threshold of 0.4 was set for the *iscontaminant* function and 70 ASVs were returned as contaminants and were consequently removed. The number of ASVs used subsequently (>0 reads and none from negative controls if occurring only here) was 2829.

Spurious ASVs defined as having <= 10 reads were identified from these 2829 and removed. These were 1161 ASVs representing 41% of the 2829 ASVs analysed here. They also represent <0.001% of the profile of any given sample. Only 7 of these 1161 ASVs were detected in >1 sample (i.e 2 samples). 1668 ASVs remain from 953 biological samples for downstream analyses (15 ASVs are unclassified at phylum level). Of these 953 samples, 78 samples were from a comparison group not enrolled in the trial hence only 875 samples are reported in this paper.

Figure S 17. Output from decontamination analysis using the DECONTAM R package. The contaminants ASVs are shown in red at the bottom right and non-contaminants in green at the top left of the plot. A total of 70 ASVs have been identified as potential contaminants and removed.

Table S 2. List of 70 ASVs detected by the DECONTAM R package as potential contaminants based on comparison between biological samples and negative controls.

Table S 3. The association between bacterial load (16S rRNA copies) and selected variables using linear mixed effects modelling.

| **Variable** | **Levels** | **^a^Number of observations (n=875)** | **Participants**  **(n=346)** | **^1^Co-efficient (95% CI)** | **^1^p value** | **^2^Adjusted Co-efficient (95% CI)** | **^2^p value** |
| --- | --- | --- | --- | --- | --- | --- | --- |
| Visit | Placebo at Week 48 | 150 (17.1%) | 150 (43.4%) | Reference | | | |
|  | AZM at Week 48 | 154 (17.6%) | 154 (44.5%) | **-0.48 [-0.65; -0.32]** | <0.0001 | **-0.46 [-0.63; -0.29]** | <0.0001 |
|  | Placebo at Week 72 | 117 (13.4%) | 117 (33.8%) | Reference | | | |
|  | AZM at Week 72 | 123 (14.1%) | 123 (35.5%) | -0.17 [-0.35; 0.02] | 0.08 | -0.19 [-0.38; 0.0] | 0.051 |
| Adherence | Adherent | 661 (75.5%) | 246 (71.1%) | Reference | | | |
|  | Non-adherent | 214 (24.5%) | 214 (61.8%) | **-0.22 [-0.37; -0.07]** | 0.004 | -0.1 [-0.26; 0.06] | 0.24 |
| Site | Malawi | 233 (26.6%) | 106 (30.6%) | Reference | | | |
|  | Zimbabwe | 642 (73.4%) | 240 (69.4%) | **0.38 [0.24; 0.52]** | <0.0001 | **0.3 [0.11; 0.49]** | 0.003 |
| Age in years |  | 779 (89%) | 346 (100%) | **0.02 [0; 0.04]** | 0.05 |  |  |
| Sex | Female | 420 (48%) | 170 (49.1%) | Reference | | | |
|  | Male | 455 (52%) | 176 (50.9%) | -0.06 [-0.19; 0.08] | 0.41 | -0.08 [-0.21; 0.05] | 0.23 |
| Season of sampling | May-Oct-Dry | 465 (53.1%) | 295 (85.3%) | Reference | | | |
|  | Nov-Apr-Rainy | 409 (46.7%) | 277 (80.1%) | 0.02 [-0.07; 0.12] | 0.60 | 0.04 [-0.06; 0.13] | 0.41 |
| MRC dyspnoea score at Baseline | 1 | 479 (54.7%) | 184 (53.2%) | Reference | | | |
|  | 2 | 316 (36.1%) | 126 (36.4%) | **-0.2 [-0.34; -0.06]** | 0.02 | -0.07 [-0.22; 0.09] | 0.76 |
|  | 3 | 53 (6.1%) | 23 (6.6%) | **-0.28 [-0.55; 0]** |  | -0.04 [-0.36; 0.28] |  |
|  | 4 | 23 (2.6%) | 11 (3.2%) | -0.36 [-0.75; 0.04] |  | -0.18 [-0.62; 0.27] |  |
|  | 5 | 4 (0.5%) | 2 (0.6%) | 0.12 [-0.81; 1.05] |  | -0.79 [-2.29; 0.71] |  |
| FEV1z |  | 862 (98.5%) | 346 (100%) | **-0.11 [-0.18; -0.04]** | 0.003 | **-0.09 [-0.16; -0.02]** | 0.02 |
| Forced Vital Capacity (FVC) |  | 852 (97.4%) | 345 (99.7%) | 0.02 [-0.08; 0.12] | 0.76 |  |  |
| FVCz |  | 852 (97.4%) | 345 (99.7%) | **-0.08 [-0.14; -0.02]** | 0.01 |  |  |
| FEV1/FVCz |  | 852 (97.4%) | 345 (99.7%) | -0.03 [-0.08; 0.02] | 0.28 |  |  |
| % Predicted FVC |  | 852 (97.4%) | 345 (99.7%) | **-0.01 [-0.01; 0]** | 0.01 |  |  |
| % Predicted FEV |  | 862 (98.5%) | 346 (100%) | **-0.01 [-0.01; 0]** | 0.003 |  |  |
| BMI-for-age *z*-score |  | 868 (99.2%) | 346 (100%) | -0.03 [-0.09; 0.02] | 0.21 |  |  |
| Weight-for-age *z*-score |  | 868 (99.2%) | 346 (100%) | -0.01 [-0.06; 0.03] | 0.61 |  |  |
| Height-for-age *z*-score |  | 868 (99.2%) | 346 (100%) | 0.02 [-0.04; 0.07] | 0.55 |  |  |
| CD4 at enrolment |  | 868 (99.2%) | 346 (100%) | 0 [0; 0] | 0.14 |  |  |
| CD4 at all visits |  | 631 (72.1%) | 346 (100%) | **0 [0; 0]** | 0.04 |  |  |
| Viral load at all visits |  | 676 (77.3%) | 343 (99.1%) | 0 [0; 0] | 0.89 |  |  |
| Viral load suppression at baseline | Suppressed | 495 (56.5%) | 193 (55.8%) | Reference | | | |
|  | Unsuppressed (≥ 1000 copies/ μl) | 377 (43.0%) | 151 (43.6%) | 0.09 [-0.04; 0.22] | 0.18 | 0.1 [-0.04; 0.23] | 0.16 |
| Weight_for_age z score | Not underweight | 407 (46.5%) | 166 (48%) | Reference | | | |
|  | Underweight | 468 (53.5%) | 180 (52%) | -0.04 [-0.17; 0.09] | 0.54 |  |  |
| Height_for_age z score | Not stunted | 434 (49.6% ) | 171 (49.4%) | Reference | | | |
|  | stunted | 441 ( 50.4%) | 175(50.6%) | -0.02 [-0.15; 0.12] | 0.81 | 0.01 [-0.12; 0.15] | 0.84 |
| Acute exacerbation during intervention | No | 752 (85.9%) | 300 (86.7%) | Reference | | | |
|  | Yes | 123 (14.1%) | 46 (13.3%) | 0.15 [-0.04; 0.34] | 0.13 |  |  |
| Hospitalised during intervention | No | 852 (97.4%) | 336 (97.1%) | Reference | | | |
|  | Yes | 23 (2.6%) | 10 (2.9%) | 0.14 [-0.27; 0.54] | 0.5 |  |  |
| Additional antibiotics during intervention | No | 843 (96.3%) | 334 (96.5%) | Reference | | | |
|  | Yes | 32 (3.7%) | 12 (3.5%) | -0.2 [-0.55; 0.15] | 0.27 |  |  |
| ^3^Any events during intervention | No | 724 (82.7%) | 288 (83.2%) | Reference | | | |
|  | Yes | 151 (17.3%) | 58 (16.8%) | 0.1 [-0.08; 0.27] | 0.28 |  |  |
| Cotrimoxazole prophylaxis at Baseline | No | 88 (10.1%) | 32 (9.2%) | Reference | | | |
|  | Yes | 783 (89.5%) | 312 (90.2%) | -0.19 [-0.41; 0.04] | 0.10 | -0.14 [-0.35; 0.07] | 0.20 |
| Age group at Baseline | 17-19y | 258 (29.5%) | 103 (29.8%) | Reference | | | |
|  | 13-16y | 374 (42.7%) | 149 (43.1%) | -0.05 [-0.21; 0.10] | 0.02 | -0.01 [-0.25; 0.24] | 0.55 |
|  | 10-12y | 165 (18.9%) | 63 (18.2%) | **-0.3 [-0.49; -0.11]** |  | -0.14 [-0.34; 0.06] |  |
|  | 6-9y | 78 (8.9%) | 31 (9%) | -0.01 [-0.34; 0.15] |  | -0.01 [-0.16; 0.15] |  |
| Ever admitted for chest problems in the past year before enrolment | No | 863 (98.6%) | 340 (98.3%) | Reference | | | |
|  | Yes | 12 (1.4%) | 6 (1.7) | -0.08 [-0.62; 0.46] | 0.76 | -0.33 [-0.97; 0.3] | 0.33 |
| Ever treated for tuberculosis before enrolment | No | 609 (69.8%) | 248 (71.9%) | Reference | | | |
|  | Yes | 263 (30.2%) | 97 (28.1%) | **0.27 [0.13; 0.41]** | <0.0001 | **0.17 [0.03; 0.32]** | 0.02 |
| Duration of ART at Baseline | 6m-2y | 80 (9.1%) | 33 (9.5%) | Reference | | | |
|  | 2-<4y | 141 (16.1%) | 58 (16.8%) | 0.16 [-0.11; 0.43] | 0.70 | -0.04 [-0.3; 0.23] | 0.71 |
|  | 4y-<6y | 187 (21.4%) | 72 (20.8%) | 0.12 [-0.14; 0.38] |  | -0.05 [-0.3; 0.21] |  |
|  | 6y+ | 442 (50.5%) | 172 (49.7%) | 0.11 [-0.13; 0.34] |  | -0.11 [-0.35; 0.12] |  |

Abbreviations: forced vital capacity (FVC), forced vital capacity z-score (FVCz), forced expiratory volume in 1 second (FEV1) z-score (FEV1z), FEV1 percentage predicted (FEVpcpred), FVC percentage predicted (FVCpcpred) and FEV1/FVCz ratio of FEV1 and FVC z-score, Body mass index (BMI). ^a^The difference between the number of observations and the total (875) represent number of missing observations for that variable. ^1^The estimate of coefficient with 95% confidence intervals and p values were obtained from univariate linear mixed effect model with participant included as a random effect and each variable and trial arm: visit interaction term as explanatory variables and log_10_ 16S rRNA copies of the sputum samples as dependent variable. ^2^The estimate of coefficient with 95% confidence intervals and p values were obtained from multivariate linear mixed effect model with participant included as a random effect, trial arm, visit and trial arm: visit interaction term and all variables that have values under the “Adjusted Coefficient” column as explanatory variables and log_10_ 16S rRNA copies of the sputum samples as dependent variable. FEV1/FVC*z*, FVC*z*, FVCpcpred, FEVpcpred were excluded from the final model because of collinearity. Viral load and CD4 counts were excluded from the final model because data was not collected at 72 weeks, the values at baseline were used instead. ^3^Any event refers to either acute respiratory exacerbation; additional antibiotics other than interventional drug or cotrimoxazole, or hospitalisation during intervention.

Table S 4. The association between Shannon diversity indices and selected variables using linear mixed effects modelling.

| **Variable** | **Levels** | **^a^Number of observations (n=875)** | **Participants**  **(n=346)** | **^1^Co-efficient (95% CI)** | **^1^p value** | **^2^Adjusted Co-efficient (95% CI)** | **^2^p value** |
| --- | --- | --- | --- | --- | --- | --- | --- |
| Visit | Placebo at Week 48 | 150 (17.1%) | 150 (43.4%) | Reference | | | |
|  | AZM at Week 48 | 154 (17.6%) | 154 (44.5%) | **0.28 [0.11; 0.45]** | 0.001 | **0.25 [0.07; 0.42]** | 0.01 |
|  | Placebo at Week 72 | 117 (13.4%) | 117 (33.8%) | Reference | | | |
|  | AZM at Week 72 | 123 (14.1%) | 123 (35.5%) | **0.20 [0.01; 0.39]** | 0.04 | **0.2 [0.01; 0.40]** | 0.04 |
| Adherence | Adherent | 661 (75.5%) | 246 (71.1%) | Reference | | | |
|  | Non-adherent | 214 (24.5%) | 214 (61.8%) | 0.02 [-0.15; 0.18] | 0.85 | 0.07 [-0.1; 0.24] | 0.42 |
| Site | Malawi | 233 (26.6%) | 106 (30.6%) | Reference | | | |
|  | Zimbabwe | 642 (73.4%) | 240 (69.4%) | 0.07 [-0.09; 0.23] | 0.39 | **0.27 [0.06; 0.47]** | 0.01 |
| Age in years |  | 779 (89%) | 346 (100%) | -0.02 [-0.04; 0] | 0.09 |  |  |
| Sex | Female | 420 (48%) | 170 (49.1%) | Reference | | | |
|  | Male | 455 (52%) | 176 (50.9%) | 0.14 [-0.01; 0.28] | 0.06 | 0.13 [-0.01; 0.27] | 0.07 |
| Season of sampling | May-Oct-Dry | 465 (53.1%) | 295 (85.3%) | Reference | | | |
|  | Nov-Apr-Rainy | 409 (46.7%) | 277 (80.1%) | -0.06 [-0.16; 0.03] | 0.20 | -0.09 [-0.19; 0.01] | 0.07 |
| MRC dyspnoea score at Baseline | 1 | 479 (54.7%) | 184 (53.2%) | Reference | | | |
|  | 2 | 316 (36.1%) | 126 (36.4%) | 0.14 [-0.01; 0.29] | 0.04 | **0.26 [0.1; 0.42]** | 0.04 |
|  | 3 | 53 (6.1%) | 23 (6.6%) | 0.03 [-0.26; 0.33] |  | 0.16 [-0.18; 0.49] |  |
|  | 4 | 23 (2.6%) | 11 (3.2%) | -0.11 [-0.53; 0.31] |  | 0.25 [-0.21; 0.71] |  |
|  | 5 | 4 (0.5%) | 2 (0.6%) | **-1.21 [-2.21; -0.21]** |  | 0.52 [-1.05; 2.09] |  |
| FEV1z |  | 862 (98.5%) | 346 (100%) | **0.21 [0.14; 0.29]** | <0.001 | **0.19 [0.12; 0.27]** | <0.001 |
| Forced Vital Capacity (FVC) |  | 852 (97.4%) | 345 (99.7%) | 0.05 [-0.06; 0.16] | 0.37 |  |  |
| FVCz |  | 852 (97.4%) | 345 (99.7%) | **0.1 [0.03; 0.16]** | 0.004 |  |  |
| FEV1/FVCz |  | 852 (97.4%) | 345 (99.7%) | **0.12 [0.06; 0.18]** | <0.001 |  |  |
| % Predicted FVC |  | 852 (97.4%) | 345 (99.7%) | **0.01 [0; 0.01]** | 0.003 |  |  |
| % Predicted FEV |  | 862 (98.5%) | 346 (100%) | **0.02 [0.01; 0.02]** | <0.001 |  |  |
| BMI-for-age *z*-score |  | 868 (99.2%) | 346 (100%) | 0.03 [-0.03; 0.08] | 0.35 |  |  |
| Weight-for-age *z*-score |  | 868 (99.2%) | 346 (100%) | 0.02 [-0.03; 0.07] | 0.47 |  |  |
| Height-for-age *z*-score |  | 868 (99.2%) | 346 (100%) | 0 [-0.06; 0.06] | 0.98 |  |  |
| CD4 at enrolment |  | 868 (99.2%) | 346 (100%) | 0 [0; 0] | 0.06 |  |  |
| CD4 at all visits |  | 631 (72.1%) | 346 (100%) | **0 [0; 0]** | 0.01 |  |  |
| Viral load at all visits |  | 676 (77.3%) | 343 (99.1%) | **0 [0; 0]** | 0.04 |  |  |
| Viral load suppression at baseline | Suppressed | 495 (56.5%) | 193 (55.8%) | Reference | | | |
|  | Unsuppressed (≥ 1000 copies/ μl) | 377 (43.0%) | 151 (43.6%) | -0.11 [-0.26; 0.03] | 0.12 | -0.06 [-0.2; 0.08] | 0.40 |
| Weight_for_age z score | Not underweight | 407 (46.5%) | 166 (48%) | Reference | | | |
|  | Underweight | 468 (53.5%) | 180 (52%) | 0.04 [-0.1; 0.18] | 0.59 |  |  |
| Height_for_age z score | Not stunted | 434 (49.6%) | 171 (49.4%) | Reference | | | |
|  | stunted | 441 (50.4%) | 175(50.6%) | 0.04 [-0.1; 0.18] | 0.57 | 0.09 [-0.05; 0.23] | 0.23 |
| Acute exacerbation during intervention | No | 752 (85.9%) | 300 (86.7%) | Reference | | | |
|  | Yes | 123 (14.1%) | 46 (13.3%) | **-0.22 [-0.42; -0.01]** | 0.04 |  |  |
| Hospitalised during intervention | No | 852 (97.4%) | 336 (97.1%) | Reference | | | |
|  | Yes | 23 (2.6%) | 10 (2.9%) | -0.28 [-0.72; 0.15] | 0.21 |  |  |
| Additional antibiotics during intervention | No | 843 (96.3%) | 334 (96.5%) | Reference | | | |
|  | Yes | 32 (3.7%) | 12 (3.5%) | -0.1 [-0.49; 0.28] | 0.60 |  |  |
| ^3^Any events during intervention | No | 724 (82.7%) | 288 (83.2%) | Reference | | | |
|  | Yes | 151 (17.3%) | 58 (16.8%) | **-0.23 [-0.41; -0.04]** | 0.02 | -0.16 [-0.35; 0.03] | 0.11 |
| Cotrimoxazole prophylaxis at Baseline | No | 88 (10.1%) | 32 (9.2%) | Reference | | | |
|  | Yes | 783 (89.5%) | 312 (90.2%) | -0.1 [-0.34; 0.15] | 0.44 | -0.13 [-0.36; 0.09] | 0.26 |
| Oxygen Saturation | Normal | 840 (96.0%) | 345 (99.7%) | Reference | | | |
|  | Abnormal | 26 (3.0%) | 26 (7.5%) | -0.27 [-0.56; 0.01] | 0.06 |  |  |
| Heart rate | Normal | 810 (92.6%) | 340 (98.3%) | Reference | | | |
|  | Abnormal | 56 (6.4%) | 44 (12.7%) | **-0.22 [-0.44; -0.01]** | 0.04 | -0.1 [-0.32; 0.11] | 0.36 |
| Age group at Baseline | 17-19y | 258 (29.5%) | 103 (29.8%) | Reference | | | |
|  | 13-16y | 374 (42.7%) | 149 (43.1%) | -0.02 [-0.02; 0.18] | 0.24 | 0.03 [-0.13; 0.2] | 0.47 |
|  | 10-12y | 165 (18.9%) | 63 (18.2%) | 0.19[-0.2; 0.40] |  | 0.17 [-0.04; 0.38] |  |
|  | 6-9y | 78 (8.9%) | 31 (9%) | 0.13 [-0.14; 0.40] |  | 0.01 [-0.24; 0.27] |  |
| Ever admitted for chest problems in the past year before enrolment | No | 863 (98.6%) | 340 (98.3%) | Reference | | | |
|  | Yes | 12 (1.4%) | 6 (1.7) | -0.14 [-0.72; 0.44] | 0.64 | 0.5 [-0.16; 1.17] | 0.16 |
| Ever treated for tuberculosis before enrolment | No | 609 (69.8%) | 248 (71.9%) | Reference | | | |
|  | Yes | 263 (30.2%) | 97 (28.1%) | **-0.24 [-0.39; -0.08]** | 0.003 | **-0.19 [-0.34; -0.04]** | 0.02 |
| Duration of ART at Baseline | 6m-2y | 80 (9.1%) | 33 (9.5%) | Reference | | | |
|  | 2-<4y | 141 (16.1%) | 58 (16.8%) | 0.07 [-0.22; 0.35] | 0.87 | 0.18 [-0.1; 0.46] | 0.63 |
|  | 4y-<6y | 187 (21.4%) | 72 (20.8%) | -0.01 [-0.28; 0.27] |  | 0.11 [-0.16; 0.37] |  |
|  | 6y+ | 442 (50.5%) | 172 (49.7%) | -0.02 [-0.27; 0.23] |  | 0.14 [-0.1; 0.39] |  |

Abbreviations: forced vital capacity (FVC), forced vital capacity z-score (FVCz), forced expiratory volume in 1 second (FEV1) z-score (FEV1z), FEV1 percentage predicted (FEVpcpred), FVC percentage predicted (FVCpcpred) and FEV1/FVCz ratio of FEV1 and FVC z-score, Body mass index (BMI). ^a^The difference between the number of observations and the total (875) represent number of missing observations for that variable. ^1^The estimate of coefficient with 95% confidence intervals and p values were obtained from linear mixed effect model with participant included as a random effect and each variable and trial arm: visit interaction term as explanatory variables and Shannon indices of the sputum samples as dependent variable. ^2^The estimate of coefficient with 95% confidence intervals and p values were obtained from multivariate linear mixed effect model with participant included as a random effect, trial arm, visit and trial arm: visit interaction term and all variables that have values under the “Adjusted Coefficient” column as explanatory variables and Shannon indices of the sputum samples as dependent variable. FEV1/FVC*z*, FVC*z*, FVCpcpred, FEVpcpred were excluded from the final model because of collinearity. Viral load and CD4 counts were excluded from the final model because data was not collected at 72 weeks, the values at baseline were used instead. ^3^Any event refers to either acute respiratory exacerbation; additional antibiotics other than interventional drug or cotrimoxazole; or hospitalisation during intervention.

### e. Alpha diversity

Figure S 18. Boxplot of Shannon alpha diversity index between trial arms at each visit (A) and between study visits in AZM (B) and Placebo (C) arms. The between trial comparisons were implemented using Wilcoxon signed rank test for unpaired samples while within-trial comparisons used Wilcoxon signed rank test for paired samples.

### f. Beta diversity- Azithromycin only

Figure S 19. Violin boxplot comparing two beta diversity metrics between samples collected from participants in the AZM arms at baseline and 48 weeks, 48 and 72 weeks and baseline and 72 weeks. PERMANOVA test used. A) Comparison of samples from baseline and 48 weeks using Aitchison distance. B) Comparison of samples from baseline and 48 weeks using Bray-Curtis distance on unrarefied ASV counts. C) Comparison of samples from 48- and 72-weeks using Aitchison distance. D) Comparison of samples from 48- and 72-weeks using Bray-Curtis distance on unrarefied ASV counts. E) Comparison of samples from baseline and 72 weeks using Aitchison distance. F) Comparison of samples from baseline and 72 weeks using Bray-Curtis distance on unrarefied ASV counts. *p values were adjusted using BH correction. The first two violin boxplots of each figure shows the distribution of the within group distances in the samples from the two visits. The third violin boxplots of each figure shows the distribution of the between group distance between the two visits. The horizontal line in the middle of the box is the median. The box presents interquartile range. The whiskers show 95% confidence interval. The shape of the violin display frequencies of values.

### g. Beta diversity- Placebo only

Figure S 20. Violin boxplot comparing two beta diversity metrics between samples collected from participants in the Placebo arms at baseline and 48 weeks, 48 and 72 weeks and baseline and 72 weeks. PERMANOVA test used. A) Comparison of samples from baseline and 48 weeks using Aitchison distance. B) Comparison of samples from baseline and 48 weeks using Bray-Curtis distance on unrarefied ASV counts. C) Comparison of samples from 48- and 72-weeks using Aitchison distance. D) Comparison of samples from 48- and 72-weeks using Bray-Curtis distance on unrarefied ASV counts. E) Comparison of samples from baseline and 72 weeks using Aitchison distance. F) Comparison of samples from baseline and 72 weeks using Bray-Curtis distance on unrarefied ASV counts. *p values were adjusted using BH correction. The first two violin boxplots of each figure shows the distribution of the within group distances in the samples from the two visits. The third violin boxplots of each figure shows the distribution of the between group distance between the two visits. The horizontal line in the middle of the box is the median. The box presents interquartile range. The whiskers show 95% confidence interval. The shape of the violin display frequencies of values.

### h. Beta diversity- Azithromycin and Placebo

Figure S 21. Principal Coordinates Analysis of Atchison (A) and Bray-Curtis (B) [on unrarefied ASV counts] distance matrixes between trial arms at each visit. The confidence ellipses define the region that contains 95% of all samples that can be drawn from the underlying “t” distribution for each arm.

### i. Relative abundance of Phyla in all samples

Figure S 22. Barplot of the relative abundances of the top 10 most prevalent phyla in all samples. Upper right, middle, and left panels show samples from participants in the AZM arm at baseline, 48 weeks and 72 weeks. The lower right, middle and lower left panels show samples from participants in the Placebo arm at baseline, 48 weeks and 72 weeks. “Others” refers all phyla that are not included in the top 10.

### j. Relative abundance of Genera in all samples

Figure S 23. Barplot of the relative abundances of the top 12 most prevalent genera in all samples. Upper right, middle, and left panels show samples from participants in the AZM arm at baseline, 48 weeks, and 72 weeks. The lower right, middle and lower left panels show samples from participants in the Placebo arm at baseline, 48 weeks, and 72 weeks. “Others” refers all other genera that are not included in the top 12.

# 2.2. Results of differential abundance of taxa testing

## 2.2.1. AZM and Placebo

### a. AZM and Placebo at baseline

No taxon was found to be differentially abundant by any of the methods.

### b. AZM and Placebo at 48 weeks

Results of all the methods are captured in Table S5 attached as a separate document.

Table S 5. Results of differential abundance testing of bacterial taxa from AZM and Placebo samples from 48 weeks using 10 methods.

Figure S 24. Heatmap displaying the q values of the genera detected as differentially abundant between AZM and placebo arms at 48 weeks by 10 statistical methods. For ANCOM2, taxa with w 0.6, 0.7, 0.8 and 0.9 were assigned q value of 0.01, 0.001, 0.0001 and 0.00001 respectively. Five genera were detected as differentially abundant by all methods (*Lautropia, Moraxella, Rothia, Treponema* and *Veilonella*).

### c. AZM and Placebo at 72 weeks.

*Treponema* was detected by Ancom2 and *Lautropia* by DESeq2 as differentially abundant taxa. None of the other methods detected a differentially abundant taxon.

Table S 6. Results of differential abundance testing of bacterial taxa from AZM and Placebo samples from 72 weeks using DESeq2.

| **Genus** | **baseMean** | **log2FoldChange** | **lfcSE** | **p value** | **Adjusted p value** |
| --- | --- | --- | --- | --- | --- |
| *Lautropia* | 149.33 | -1.42 | 0.35 | 1.44E-06 | 0.0002 |

Table S 7. Results of differential abundance testing of bacterial taxa from AZM and Placebo samples from 72 weeks using Ancom-II

| **Genus** | **W** | **detected_0.9** | **detected_0.8** | **detected_0.7** | **detected_0.6** |
| --- | --- | --- | --- | --- | --- |
| *Treponema* | 41 | FALSE | TRUE | TRUE | TRUE |

## 2.2.2. Azithromycin arm only

### a. AZM at baseline and 48 weeks

Results of all the methods are captured in Table S8 attached as a separate document.

Table S 8. Results of differential abundance testing of bacterial taxa from samples from the AZM arm at baseline and 48 72 weeks using 10 methods.

Figure S 25. Heatmap displaying the q values of the genera detected as differentially abundant within the AZM arm between baseline and 48-week samples by 10 methods. For ANCOM2, taxa with w 0.6, 0.7, 0.8 and 0.9 were assigned q value of 0.01, 0.001, 0.0001 and 0.00001 respectively. *Lautropia, Moraxella, Treponema, Oribacterium, F0058*, and ASV 209 were detected as differentially abundant by all methods.

### b. AZM at 48 and 72 weeks

Results of all the methods are captured in Table S9 attached as a separate document.

Table S 9. Results of differential abundance testing of bacterial taxa from samples from the AZM arm at 48 and 72 weeks using 10 methods.

Figure S 26. Heatmap displaying the q values of the genera detected as differentially abundant within the AZM arm between 48- and 72-week samples by 10 methods. For ANCOM2, taxa with w 0.6, 0.7, 0.8 and 0.9 were assigned q value of 0.01, 0.001, 0.0001 and 0.00001 respectively. Only *Moraxella* was detected as differentially abundant by all methods.

### c. AZM at baseline and 72 weeks

No taxon was found to be differentially abundant by any of the methods.

## 2.2.3. Placebo arm only

a. Placebo at baseline and 48 weeks

No taxon was found to be differentially abundant by any of the methods.

### b. Placebo at 48 and 72 weeks

Moraxella was detected as differentially abundant by DESeq2. None of the other methods detected a differentially abundant taxon.

Table S 10. Results of differential abundance testing of bacterial taxa from Placebo samples from 48 and 72 weeks using DESeq2.

| **Genus** | **baseMean** | **log2FoldChange** | **lfcSE** | **p value** | **Adjusted p value** |
| --- | --- | --- | --- | --- | --- |
| *Moraxella* | 242.26 | -9.05E-06 | 0.001 | 9.54E-07 | 1.31E-04 |

### c. Placebo at baseline and 72 weeks

Moraxella detected by DESeq2. None of the other methods detected a differentially abundant taxon.

Table S 11. Results of differential abundance testing of bacterial taxa from Placebo samples from baseline and 72 weeks using DESeq2.

| **Genus** | **baseMean** | **log2FoldChange** | **lfcSE** | **p value** | **Adjusted p value** |
| --- | --- | --- | --- | --- | --- |
| *Moraxella* | 231.49 | -2.42 | 0.59 | 3.82E-05 | 5.20E-03 |

# 2.3. Results of SIMPER analysis

Table S 12. Contributions of top genera to overall dissimilarity between AZM and Placebo arms at 48 weeks and, within the AZM arm, between Baseline and 48-week samples- SIMPER analysis.

| **Genus** | **AZM and Placebo at 48 weeks** | | | | | | **Baseline and 48 weeks in the AZM arm** | | | | | |
| --- | --- | --- | --- | --- | --- | --- | --- | --- | --- | --- | --- | --- |
|  | **Average contribution to overall dissimilarity.** | **Standard deviation of contribution.** | **Mean abundance in AZM arm** | **Mean abundance in Placebo arm** | **Ordered cumulative contribution** | ***P value** | **Average contribution to overall dissimilarity.** | **Standard deviation of contribution.** | **Mean abundance at Baseline** | **Mean abundance at 48 weeks** | **Ordered cumulative contribution** | ***P value** |
| *Haemophilus* | 12.8 | 14.2 | 28.4 | 17.5 | 23.6 | 0.003 | 11.4 | 13.2 | 25.8 | 17.9 | 22.6 | 0.59 |
| *Neisseria* | 8.4 | 6.3 | 17.7 | 20.5 | 39.2 | 0.15 | 8.1 | 6.2 | 19.1 | 20.3 | 38.7 | 0.01 |
| *Streptococcus* | 6.2 | 4.8 | 15.3 | 19.6 | 50.7 | 0.002 | 5.8 | 4.8 | 15.9 | 19.4 | 50.2 | 0.004 |
| *Prevotella* | 5 | 4.4 | 8.1 | 10 | 59.9 | 0.07 | 5 | 4.3 | 9.4 | 10.1 | 60.1 | 0.73 |
| *Moraxella* | 2.4 | 8 | 4.1 | 0.9 | 64.4 | <0.0001 | 1.4 | 5 | 1.9 | 1 | 62.9 | 0.17 |
| *Veillonella* | 2.3 | 1.9 | 3.4 | 5.4 | 68.7 | <0.0001 | 2.1 | 1.8 | 3.8 | 5.3 | 67.2 | <0.001 |
| *Porphyromonas* | 1.8 | 2.7 | 2.9 | 3 | 72 | 0.10 | 1.7 | 2.2 | 3.1 | 3.1 | 70.5 | 0.06 |
| *Fusobacterium* | 1.7 | 2.4 | 3.5 | 2.2 | 75.2 | 0.10 | 1.8 | 2.5 | 3.6 | 2.2 | 74 | 0.002 |
| *Leptotrichia* | 1.5 | 2.6 | 2 | 2.2 | 78 | <0.0001 | 1.5 | 2.5 | 2 | 2.2 | 76.9 | <0.0001 |
| *Actinobacillus* | 1.4 | 2.5 | 1.8 | 1.7 | 80.6 | 0.22 | 1.3 | 2.2 | 1.6 | 1.7 | 79.4 | 0.06 |
| *Lautropia* | 1.4 | 2 | 0.8 | 2.9 | 83.3 | 0.01 | 1.4 | 2 | 1.1 | 2.9 | 82.3 | 0.88 |
| *Rothia* | 1.3 | 1.4 | 1.6 | 2.8 | 85.7 | 0.73 | 1.2 | 1.4 | 1.4 | 2.8 | 84.8 | 0.57 |
| *Alloprevotella* | 1.3 | 1.3 | 2 | 2.3 | 88.1 | 0.83 | 1.6 | 1.5 | 3.1 | 2.2 | 87.9 | 0.64 |
| *Actinomyces* | 0.6 | 0.8 | 0.7 | 1.1 | 89.1 | <0.001 |  | | | | | |
| *Granulicatella* | 0.6 | 0.5 | 1.1 | 1.5 | 90.2 | <0.001 |  |  |  |  |  |  |
| *Gemella* | 0.6 | 0.6 | 1 | 1.3 | 91.2 | <0.001 |  |  |  |  |  |  |
| *Aggregatibacter* | 0.4 | 0.7 | 0.7 | 0.4 | 92.1 | 0.99 |  |  |  |  |  |  |

Contributions by genus were assessed by similarity of percentages (SIMPER) analysis of Bray-Curtis distance. Average dissimilarity is a measure of dissimilarity accounted for by each genus between the sputum bacteriome composition between trial arms at 48 weeks or, within the AZM arm, between baseline and 48 week visits. Contribution (%) is the percentage of total dissimilarity that the contribution of each genus accounts for, calculated as the mean contribution divided by mean dissimilarity across samples. Cumulative (%) is percentage of dissimilarity that is accounted for by all genera included in the model to this point. Mean abundance is the mean relative abundance of each genus. Only the taxa accounting for 92% of dissimilarity are shown. *p values for comparison of mean abundance between AZM and placebo at 48 weeks by Wilcoxon signed-rank test with BH correction.

# 2.4. Results of linear regression of within-participant change in beta diversity and lung function.

Table S 13. Univariate linear regression analysis of within-participant Aitchison distance (outcome) and within-participant change in lung function metrics (FVCz and FEV1z) between visits.

| **Trial arm** | **Within-participant change in FEV1z** | | | **Within-participant change in FVCz** | | |
| --- | --- | --- | --- | --- | --- | --- |
|  | **coef** | **stderr** | **pval** | **coef** | **stderr** | **pval** |
| AZM | 1.05 | 0.45 | 0.02 | 0.95 | 0.42 | 0.02 |
| Placebo | 0.3 | 0.57 | 0.6 | -0.71 | 0.46 | 0.13 |

Associations were tested with MaAsLin2 using a linear regression model with FEV1z or FVCz and trial arm as fixed effects and within-participant change in beta diversity measured using Aitchison’s distance as outcome. Statistical significance was corrected for multiple testing using Benjamini/Hochberg correction. Columns correspond to the within-participant change in genus, trial arm, the coefficient estimate (coef) and standard error from the model (stderr), nominal p-value (pval Number of samples in azithromycin (AZM) and placebo arms are 377 and 365 respectively.

# REFERENCES

1. Kaul A, Mandal S, Davidov O, Peddada SD. Analysis of Microbiome Data in the Presence of Excess Zeros. Front Microbiol. 2017;8:2114.

2. Fernandes AD, Reid JN, Macklaim JM, McMurrough TA, Edgell DR, Gloor GB. Unifying the analysis of high-throughput sequencing datasets: characterizing RNA-seq, 16S rRNA gene sequencing and selective growth experiments by compositional data analysis. Microbiome. 2014;2:15.

3. Love M, Anders S, Huber W. Differential analysis of count data–the DESeq2 package. Genome Biol. 2014;15:10–1186.

4. Lin H, Peddada SD. Analysis of compositions of microbiomes with bias correction. Nat Commun. 2020;11:3514.

5. Martin BD, Witten D, Willis AD. Modeling microbial abundances and dysbiosis with beta-binomial regression. Ann Appl Stat. NIH Public Access; 2020;14:94.

6. Mallick H, Rahnavard A, McIver LJ, Ma S, Zhang Y, Nguyen LH, et al. Multivariable association discovery in population-scale meta-omics studies. Coelho LP, editor. PLOS Comput Biol. 2021;17:e1009442.

7. Schneeberger PHH, Prescod J, Levy L, Hwang D, Martinu T, Coburn B. Microbiota analysis optimization for human bronchoalveolar lavage fluid. Microbiome. 2019;7:141.

8. Claassen-Weitz S, Gardner-Lubbe S, Mwaikono KS, du Toit E, Zar HJ, Nicol MP. Optimizing 16S rRNA gene profile analysis from low biomass nasopharyngeal and induced sputum specimens. BMC Microbiol. 2020;20:113–113.
